# Chiral Lanthanide Complexes with l- and d-Alanine: An X-ray and Vibrational Circular Dichroism Study

**DOI:** 10.3390/molecules25122729

**Published:** 2020-06-12

**Authors:** Krzysztof Lyczko, Joanna E. Rode, Jan Cz. Dobrowolski

**Affiliations:** Institute of Nuclear Chemistry and Technology, Dorodna 16, 03-195 Warsaw, Poland; J.Rode@ichtj.waw.pl (J.E.R.); j.dobrowolski@nil.gov.pl (J.C.D.)

**Keywords:** chiral lanthanide complexes, alanine, crystal structure, lanthanide contraction effect, VCD, DFT

## Abstract

A whole series of [Ln(H_2_O)_4_(Ala)_2_]_2_^6+^ dimeric cationic lanthanide complexes with both l- and d-alanine enantiomers was synthesized. The single-crystal X-ray diffraction at 100 and 292 K shows the formation of two types of dimers (**I** and **II**) in crystals. Between the dimer centers, the alanine molecules behave as bridging (*μ*_2_-O,O’-) and chelating bridging (*μ*_2_-O,O,O’-) ligands. The first type of bridge is present in dimers **I**, while both bridge forms can be observed in dimers **II**. The IR and vibrational circular dichroism (VCD) spectra of all l- and d-alanine complexes were registered in the 1750–1250 cm^−1^ range as KBr pellets. Despite all the studied complexes are exhibiting similar crystal structures, the spectra reveal correlations or trends with the Ln–O1 distances which exemplify the lanthanide contraction effect in the IR spectra. This is especially true for the positions and intensities of some IR bands. Unexpectedly, the ν(C=O) VCD bands are quite intense and their composed shapes reveal the inequivalence of the C=O vibrators in the unit cell which vary with the lanthanide. Unlike in the IR spectra, the ν(C=O) VCD band positions are only weakly correlated with the change of Ln and the VCD intensities at most show some trends. Nevertheless, this is the first observation of the lanthanide contraction effect in the VCD spectra. Generally, for the heavier lanthanides (Ln: Dy–Lu), the VCD band maxima are very close to each other and the mirror reflection of the band of two enantiomers is usually better than that of the lighter Lns. DFT calculations show that the higher the multiplicity the higher the stability of the system. Actually, the molecular geometry in crystals (at 100 K) is well predicted based on the highest-spin structures. Also, the simulated IR and VCD spectra strongly depend on the Ln electron configuration but the best overall agreement was reached for the Lu complex, which is the only system with a fully filled *f*-shell.

## 1. Introduction

Lanthanides are used in diverse fields of medical therapy and diagnostics [1]. They are among the best contrast enhanced agents in medical Magnetic Resonance Imaging and the Gd-complexes in particular are routinely used in clinics [2,3]. Lanthanide-containing nanomaterials have the potential to be a sensitizer in photodynamic cancer therapy [4,5,6]. Radiolanthanides are applied for radiotheranostics [7,8]. Moreover, lanthanides can replace calcium in proteins for bone tissue regeneration engineering because they have similar ionic radii and ligand specificity to Ca ions [9]. They also offer great opportunities because of their ability to induce perforation of the cell membranes [10,11,12], hydrolyze DNA and RNA [13], and scavenge free radicals [14]. Lanthanides can be exploited as diverse spectroscopic probes in living organisms and biological systems. Hence, the search for the better recognition, detection, and accessibility of chiral biomolecules with lanthanide coordination compounds is an important goal of research and innovation. Their widespread use is responsible for the presence of lanthanide in human tissues such as liver, spleen, lungs, adrenal glands, and bones [15]. Still, the understanding of the biological role of lanthanides is in its early stages and, so far, lanthanides biosafety has been little taken into account [16,17,18,19]. Some hundreds of lanthanide-complexed biomacromolecules examined based on Protein Data Bank demonstrated that lanthanides can be bound to a wide variety of protein types [20]. Nevertheless, the interactions of the lanthanide ions with large biomolecules should resemble those found in their complexes with small ligands. The first and most important lanthanide ligands in biomacromolecules are the side chain amino acid COO^−^ groups and water molecules. For that reason, the Ln compounds with amino acids are a very important goal of studies.

Hitherto, various lanthanide–amino acids coordination complexes have been structurally characterized including: Alanine [21,22,23,24,25,26,27,28], proline [29,30,31,32,33,34], glycine [35,36,37,38], valine [37,39,40], and glutamic acid [41,42,43]; and, sparingly, isoleucine [44,45], leucine [46], phenylalanine [47], tyrosine [48], cysteine, aspartic acid [21], and histidine [49]. The amino acid ligands bridge the lanthanide ions forming dimeric, polymeric, or cluster species. Syntheses of these complexes depend on reagent molar ratios and the pH of the reaction solutions.

There is a persistent interest in the chiral complexes of metals with the 4*f*^n^ electron configuration [50,51,52,53,54,55]. Nowadays, not only electronic circular dichroism (ECD) can be used for the chiroptical characterization of such species. Vibrational circular dichroism (VCD), Raman optical activity (ROA), and circularly polarized luminescence (CPL) have become accessible and powerful techniques for investigating new chiral materials, biomolecules, cells, and soft tissues. Albeit, some lanthanide complexes [56,57,58,59,60,61] have already been measured and described using chiroptical methods, to the best of our knowledge, the VCD spectra of the chiral lanthanide–amino acid complexes have never been registered and interpreted. This study is devoted to the X-ray structural description of a series of lanthanide complexes with both enantiopure forms of alanine and to the interpretation of their VCD spectra measured as solids dispersed in the KBr pellets. Until now, a few [Ln(H_2_O)_4_(Ala)_2_]_2_(ClO_4_)_6_ complexes have been synthesized and their crystal structures deposited in the Cambridge Structural Database (Ln stands for lanthanum(III) [21], neodymium(III) [22], europium(III) [24], gadolinium(III) [25], terbium(III) [26], and erbium(III) [28]). However, the enantiopure forms of alanine were used only for the Tb^3+^ and Nd^3+^ ions and the complexes were shown to crystallize in the *P*1 space group. The remaining complexes contain the racemic mixture of this amino acid and crystallize in the achiral (*P*$\bar{1}$ and *C*2/c) space groups. Additionally, the crystal structures of such complexes but with chloride or mixed perchlorate-chloride anions with the formulas [Ho(H_2_O)_4_(L-Ala)_2_]_2_Cl_6_ [27] and [Sm(H_2_O)_4_(L-Ala)_2_]_2_(ClO_4_)_4_Cl_2_ [23], both crystallizing in the chiral space group *P*1, have been obtained.

Here, we present the crystal structures and the VCD spectra for a series of dimeric lanthanide(III) complexes with l- and d-alanine enantiomers. The VCD spectra of these species have not been known before and the majority of the X-ray structures are also shown for the first time. For selected systems the experimental measurements are supported by quantum-chemical calculations over the molecular structure and vibrational spectra.

## 2. Results and Discussion

### 2.1. Synthesis

**Scheme 1:**
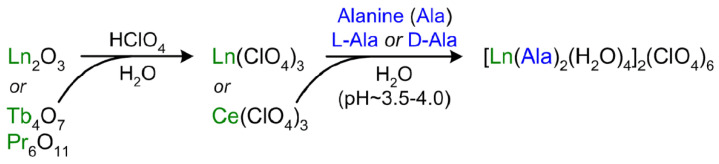
Synthesis of lanthanide complexes with alanine.

A series of dimeric complexes of 3+ charged lanthanide cations (except radioactive promethium) with both enantiomers (L- and D-forms) of alanine was obtained. The appropriate lanthanide oxides (except commercially available cerium(III) perchlorate hexahydrate) were the starting materials which were converted into perchlorate salts, and then were reacted with the amino acid in aqueous solution at pH of 3.5–4.0. A molar ratio of metal to alanine of 1:2 was applied for the lanthanides from Nd to Lu. In turn, for the remaining lightest lanthanides (La–Pr) a 1:1 molar ratio was used because with higher amounts of alanine the formation of a polymeric structure was also observed. Obtaining crystals was forced by the evaporation of a significant amount of water from the solution. A simplified reaction path towards the lanthanide complexes is shown in Scheme 1. The preparation of these compounds was inspired by the methods described earlier [22].

### 2.2. Molecular and Crystal Structures of the Prepared Complexes

**Figure 1:**
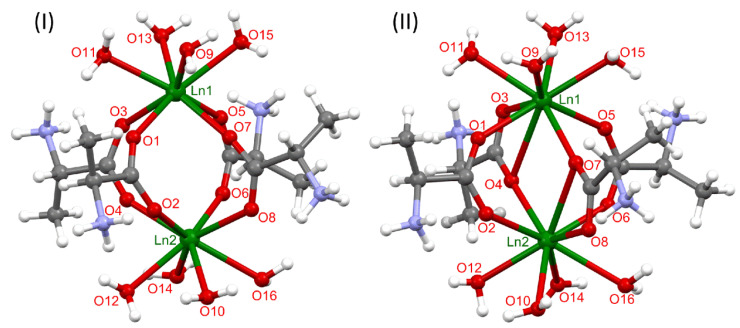
Molecular structures of cationic dimeric complexes [Ln(H_2_O)_4_(L-Ala)_2_]_2_^6+^ for heavy lanthanides from Tb to Lu (**I**) and light lanthanides from La to Nd (**II**). Perchlorate anions are omitted for clarity.

**Figure 2:**
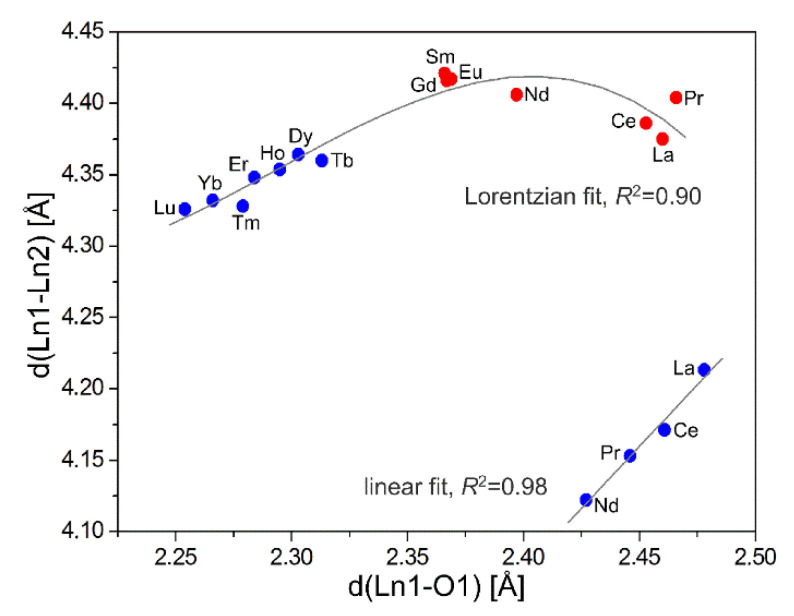
Relationship between the Ln1−O1 and Ln1···Ln2 distances determined by the X-ray diffraction measurements in 100 K (blue) and 292 K (red).

**Figure 3:**
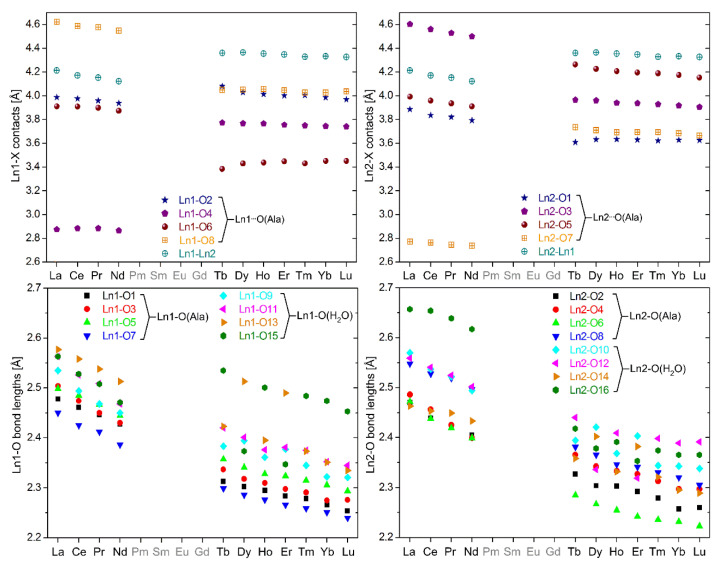
Graphical distribution of bond lengths and distances obtained from measurements at 100 K for both lanthanide atoms in dimeric complexes [Ln(H_2_O)_4_(L-Ala)_2_]_2_(ClO_4_)_6_.

The crystal structure of lanthanide complexes with both l- and d-alanine was determined by a single-crystal X-ray diffraction at a low temperature (Table 1 and Table 2, Tables S1 and S2). However, for the detailed crystal structure description only the complexes with l-alanine were taken into account. All complexes crystallize in the simplest (*P*1) space group and have a general formula [Ln(H_2_O)_4_(Ala)_2_]_2_(ClO_4_)_6_ (Ln-lanthanide; Ala-l- or d-alanine). The majority of them have a unit cell volume of *ca.* 1100 Å^3^ and one Ln-complex per unit. On the other hand, the Sm, Eu, and Gd complexes have a surprisingly large unit cell exceeding 5500 Å^3^ with five crystallographically inequivalent Ln-complexes (Table S3). Yet, the solution of these structures did not provide structural data of a quality sufficient for CSD and were omitted from further discussion. The molecular structure consists of discrete [Ln(H_2_O)_4_(Ala)_2_]_2_^6+^ cations and perchlorate anions. The compounds form characteristic dimers in which four water molecules coordinate to the metal ions and four alanine moieties bridge the two [Ln(H_2_O)_4_]^3+^ parts through carboxylate groups. The alanine ligands exist in a zwitterionic form with a deprotonated carboxyl group and a protonated amino part. In dimers, two lanthanide fragments are non-centrosymmetric due to the homochirality of the alanine used, whereas for the racemic alanine the dimer system becomes centrosymmetric [22]. Here, two different types of dimers can be distinguished (Figure 1 and Figure S1). In the case of heavier lanthanides, from terbium to lutetium, alanines form the *µ*_2_-O,O’- bridges (type **I**). For the lightest lanthanides, from lanthanum to neodymium, two alanine bridging modes are observed (type **II**): Besides the purely bridging *µ*_2_-O,O’- one a chelating bridging *µ*_2_-O,O,O’- connection also occurs. Every lanthanide ion in the dimer **I** is surrounded by eight oxygen atoms (CN = 8) in a distorted square antiprismatic geometry. In dimer **II** the additional O atom of the *µ*_2_-O,O,O’- bridge contributes to the formation of distorted mono-capped square antiprismatic polyhedron around the metal center (CN = 9). It is worth noticing that only dimer **I** was previously observed for a Nd alanine complex though Nd belongs to the light lanthanides [22]. However, the two dimeric types were found for Ln complexes with glycine [38,62]. In the *µ*_2_-O,O’-bridges, the mean metal-oxygen bond length decreases from La to Lu (except subsequent Pm, Sm, Eu, and Gd) and is equal to 2.488, 2.463, 2.445, 2.424, 2.333, 2.316, 2.306, 2.297, 2.288, 2.276, and 2.269 Å, respectively. The same trend is observed for the mean Ln−O_(H2O)_ bond lengths: 2.561 (La), 2.536 (Ce), 2.520 (Pr), 2.494 (Nd), 2.421 (Tb), 2.402 (Dy), 2.392 (Ho), 2.382 (Er), 2.377 (Tm), 2.361 (Yb), and 2.355 Å (Lu). The above tendencies remain in agreement with the lanthanide contraction effect [63]. Moreover, in every dimeric compound the mean Ln−O_(Ala)_ bond length is shorter than the mean Ln−O_(H2O)_ distance. The additional bridging bond lengths found in dimers of the lightest lanthanides (La–Nd) (Ln1−O4 and Ln2−O7, Figure 1), responsible for the formation of *µ*_2_-O,O,O’- connections, are in the range of 2.736(5)−2.885(5) Å. For heavier lanthanides, the shortest distances of that type (Ln1···O6) are about 0.6 Å longer (vary from 3.383(6) to 3.452(5) Å). Furthermore, the dimers **II** are characterized by the close distance between O atoms of chelating bridging alanine groups (O4···O7) with a mean value of 3.211 Å (range from 3.183(7) to 3.231(6) Å). In turn, the similar shortest O···O contacts in the dimers **I** are much longer and equal meanly to 4.025 (O1···O6) and 4.401 Å (O4···O7). The presence of two types of dimers also affects the metal–metal distances (Table S4). The Ln···Ln contacts for the first four lightest lanthanides change from 4.213(1) Å (La) to 4.122(1) Å (Nd) and are a bit shorter than in the case of heavier lanthanides which fall in a range of 4.326(1)−4.364(1) Å. The clear difference between both types of dimers’ geometry (Figure 2) shows the relationship between the selected Ln−O bond length (namely Ln1−O1) and the metal–metal distance in the l-alanine complexes. For the dimeric lanthanide complexes with l-alanine, the selected bond lengths and distances are compared in detail in Table S4 and their graphical distribution is presented in Figure 3. The geometrical parameters for the corresponding d-alanine complexes show very similar relationships (Table S5 and Figure S2).

In all studied complexes the crystal packing is stabilized by the N−H···O_(ClO4)_, O−H_(H2O)_···O_(ClO4)_ and O−H_(H2O)_···O_(H2O)_ interactions.

The known crystal structure of [Nd(H_2_O)_4_(L-Ala)_2_]_2_(ClO_4_)_6_ measured at room temperature [22] exhibited the presence of type **II** dimers. In contrast, the same complex measured by us in 100 K has the dimer of type **I**. This prompted us to carry out additional X-ray measurements at an ambient temperature for the complexes with dimer **II** structures (Table S6). Thus, for the same complexes of La, Ce, Pr, and Nd measurements were first carried out at 292 and then at 100 K. We found that within this temperature range, the crystals undergo a subtle structural transition. It turns out that for the lightest lanthanides a very low temperature stabilizes dimer **II**, while, at room temperature, they exhibit a structure similar to dimer **I**. With a significant decrease in temperature, the Ln1···O4 and Ln2···O7 distances are shortened, transforming the *µ*_2_-O,O’- bridges into *µ*_2_-O,O,O’- bridging connections (Table S7). Moreover, the measurements at 292 K were extended to the l-alanine complexes of Sm, Eu, and Gd. In these three cases, in contrast to the low temperature measurements, only a smaller unit cell of *ca.* 1150 Å^3^ with Z = 1 was obtained. These complexes also demonstrate a dimer **I** structure similar to the remaining heavy lanthanides complexes. The selected geometrical parameters received from room temperature measurements are collected in Table S8. Their graphical distribution (Figure S3) shows a weaker correlation between bond lengths and Ln than that to be found in the low temperature data shown in Figure 3 and Figure S2.

### 2.3. Experimental IR and VCD Spectra of the Studied Complexes

**Figure 4:**
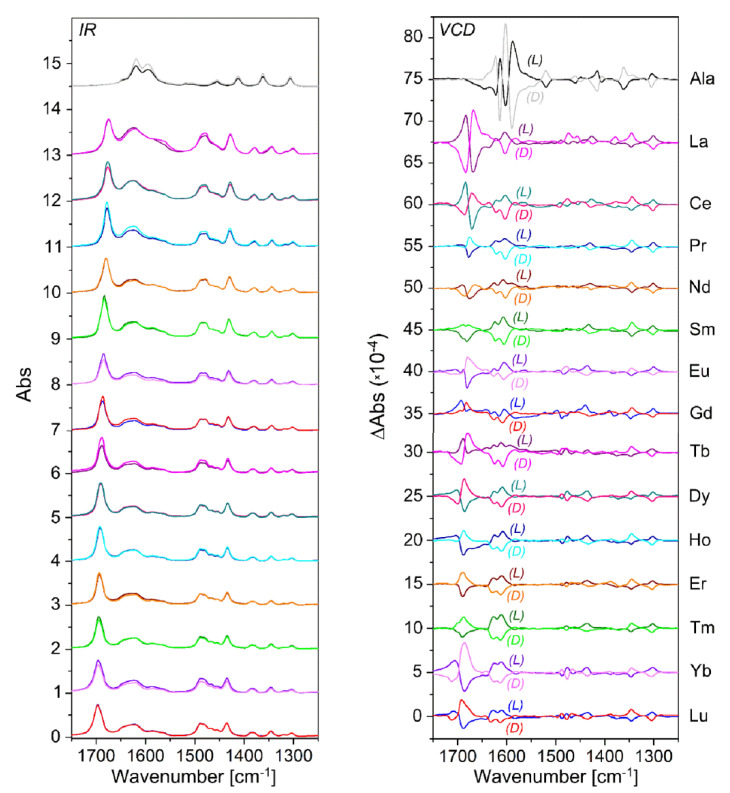
IR and vibrational circular dichroism (VCD) spectra of solid dimeric lanthanide complexes with l- and d-alanine in comparison with pure enantiomeric forms of this amino acid (measured in KBr pellets).

The IR and VCD spectra of all [Ln(H_2_O)_4_(L-Ala)_2_]_2_(ClO_4_)_6_ compounds and their D-enantiomers were registered in the 1750−1250 cm^−1^ range as KBr pellets (Figure 4). Additionally, the spectra are juxtaposed with the solid-state spectra of alanine enantiomers (Figure 4). Generally, the VCD spectra of the enantiomeric complexes display mirror symmetry. In some spectral ranges, for the Nd, Gd, and Tb complexes the mirror symmetry is not perfect. At first glance, the IR and VCD spectra of different metal complexes do not differ significantly. Indeed, all spectra are taken at room temperature at which all studied complexes exhibit similar structures. However, a deeper study of the spectra allows us to find some specific correlations or trends between the spectral features and the Ln1−O1 bond lengths. With this aim, the spectra of both L- and D-forms were normalized to the most intense ν(C=O) IR band, set as 1. The normalization factors were next transferred to the VCD bands.

**Figure 5:**
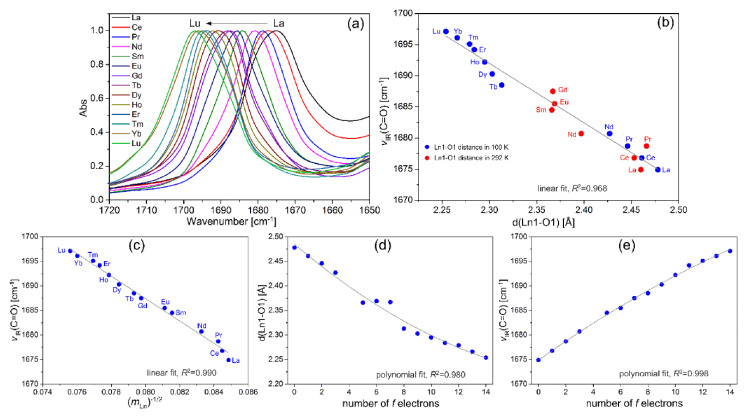
(**a**) The intensity-normalized ν(C=O) IR bands; the linear correlations between the ν(C=O) IR band frequency and: (**b**) The Ln1−O1 distance in the Ln complexes with l-alanine, and (**c**) the inverse square root of the lanthanide atomic mass (*m*)^−1/2^; the slightly non-linear correlations demonstrating the lanthanide contraction effect in: (**d**) The Ln1−O1 distance, and (**e**) the ν(C=O) frequency.

The ν(C=O) stretching vibrations band positioned at *ca.* 1690 cm^−1^ is at its most intense in the mid IR range. The band position varies within *ca.* 20 cm^−1^ and a linear correlation with the Ln1−O1 bond length reveals a relationship between the vibrational spectroscopy and the molecular structure of the Ln complexes (Figure 5a,b). Surprisingly, the heavier the Ln-atom the higher the frequency. Note, that within the two-atom-oscillator approximation, a correlation of ν(C=O) frequency with (m_Ln_)*^−^*^1/2^ is expected (where m_Ln_ is the lanthanide atomic mass). Here, this is really the case (Figure 5c). The correlation is significantly stronger than that against the Ln−O distance (Figure 5b), because the former depends precisely on well determined atomic masses while the latter is additionally dependent on non-systematic factors related to the intra- and intermolecular interactions in the crystal structures. However, despite the dependence on m_Ln_, the stretching Ln−O vibration force constants k_Ln-O_ were also shown to linearly correlate with the Ln−O bond lengths [64]. The rationale for the “anomalous” correlation (Figure 5b) is in the lanthanide contraction effect (Figure 5d,e) which converts the slope of the tendency. The contraction effect occurs because of incomplete shielding of the outer orbitals by the 4*f* electrons, which causes the effective charge of the Ln nuclei is increased and in consequence the corresponding ionic radii and the Ln−O distances are decreased [63,65,66,67]. Thus, the decrease of the d(Ln1−O1) distances is first of all a result of the decrease of the Ln radii. Such a clear lanthanide effect in vibrational spectroscopy is not very often reported (e.g., [64,68,69,70,71]), therefore, hereafter, whenever possible, we show correlation with the number of the 4*f* electrons instead of the Ln−O distance, which in these cases always is taking place, too.

**Figure 6:**
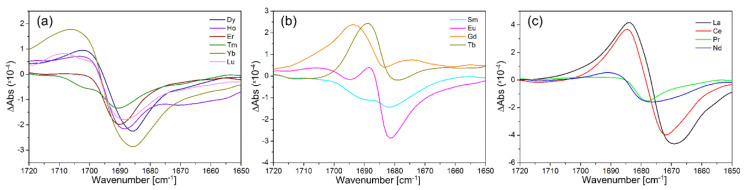
The +/− pattern of the ν(C=O) VCD bands in the Ln complexes with l-alanine reveals variations with the slight changes in the Ln1−O1 distance shown in Figure 5. Systems with the Ln1−O1 distance: (**a**) smaller than 2.30 Å, (**b**) between 2.30 and 2.40 Å, and (**c**) greater than 2.40 Å.

**Figure 7:**
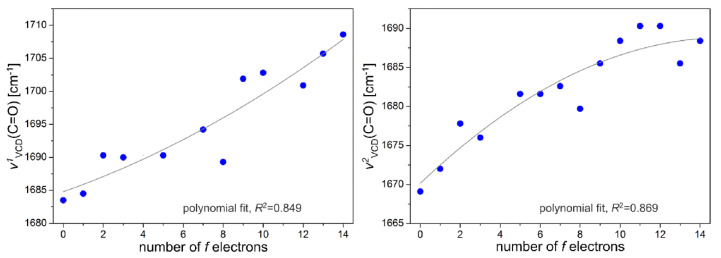
Changes of the ν^1^(C=O) and ν^2^(C=O) VCD maxima with the number of 4*f* electrons in the Ln complexes with l-alanine.

Moreover, the ν(C=O) stretching vibrations band shape varies with the Ln ion (Figure S4). In the uncomplexed alanine, the band is split into antisymmetric and symmetric components at 1620 and 1596 cm^−1^, respectively. Unlike in the solution [72,73,74,75], and in line with our previous investigations [76], and other new findings [77], the VCD spectra show the presence of quite intense ν(C=O) bands (Figure 4), despite the fact that they are *ca.* 10^4^ times weaker than the IR ones. The ν(C=O) VCD pattern is not homogenous. Generally, there are two ν(C=O) VCD bands, at *ca.* 1700 and 1680 cm^–1^: the high-frequency one is mainly positive but can be also negative or can disappear, while the other is always negative. Thus, the +/− pattern is the most common and it is observed for La, Ce, Tb, Dy, Yb, and Lu l-alanine complexes (Figure 6 and Figure S4). A similar pattern is registered for Nd and Gd crystals, however, the band maxima are shifted with respect to each other for L-Ala and D-Ala complexes. Yet, the −/− bands’ sequence also occurs for Sm and Tm, while one well-developed negative VCD band is recorded for the Pr, Eu, and Er complexes with l-alanine (Figure 6 and Figure S4). Thus, the ν(C=O) VCD bands exhibit a complex nature demonstrating both antisymmetric and symmetric vibrations and an inequivalence of the two C=O vibrators in the molecule (Figure 4). While the ν(C=O) VCD positions exhibit a fair correlations revealing, probably the first example of the lanthanide contraction effect in the VCD spectra (Figure 7), the VCD band intensity changes do not show any regularities (Figure S5).

**Figure 8:**
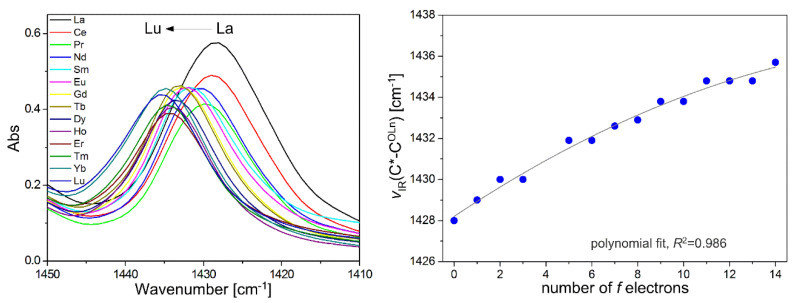
The ν(C*−C^OLn^) IR bands (left) and linear correlation between the ν(C*−C^OLn^) IR band maximum and the number of 4*f* electrons (right) in the Ln complexes with l-alanine. The IR intensity in all spectral regions was normalized to the ν(C=O) band (Figure 5a).

**Figure 9:**
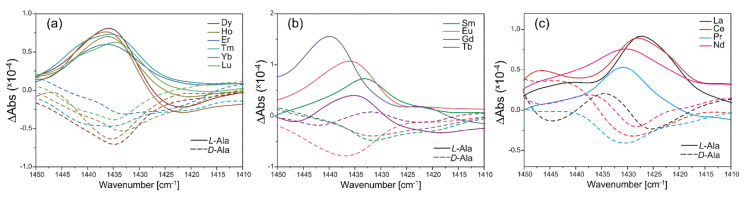
The VCD spectral pattern of the ν(C*−C^OLn^) bands in the Ln complexes with l- and d-alanine reveals variations with the slight changes in the Ln−O1 distance shown in Figure 5. Systems with the Ln1−O1 bond length: (**a**) Smaller than 2.30 Å, (**b**) between 2.30 and 2.40 Å, and (**c**) greater than 2.40 Å.

The position of the ν(C*−C^OLn^) stretching vibrations IR band at *ca.* 1430 cm*^−^*^1^ (assigned by the calculations, see Section 2.4) is linearly increasing with the number of 4*f* electrons as well (Figure 8). Although, for the heavy lanthanides (Ln: Dy–Lu), the maxima of the ν(C*−C^OLn^) VCD bands are positioned at very similar wavenumbers and are well mirror-reflected, for the remaining lanthanides the IR and VCD band positions change noticeably (Figure 9). It is worth noting that, for the lighter lanthanides, the VCD baseline was not stable, the VCD intensities for l- and d-alanine complexes were unequal, and a regular correlation for the VCD intensities of the ν(C*−C^OLn^) band was hardly possible.

**Figure 10:**
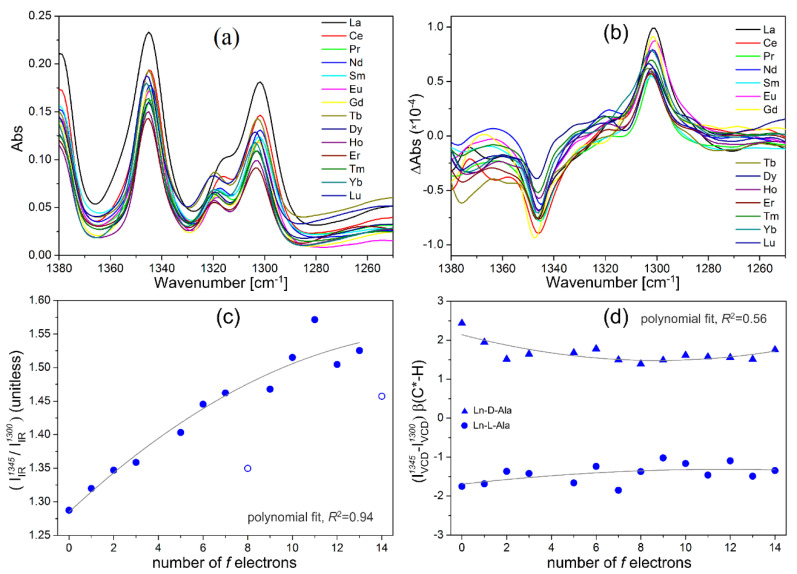
Juxtaposition of the IR (**a**) and VCD (**b**) spectral patterns for the β(C*−H) bending vibration bands occurring in the Ln complexes with l- and d-alanine. (**c**) The lanthanide contraction effect on the IR intensity ratio for the β(C*−H) bending vibration bands at *ca.* 1345 and 1300 cm^−1^ (the open circles denote points excluded from correlations). (**d**) The lanthanide contraction effect on the VCD intensity trends of the β(C*−H) bending vibration bands.

**Figure 11:**
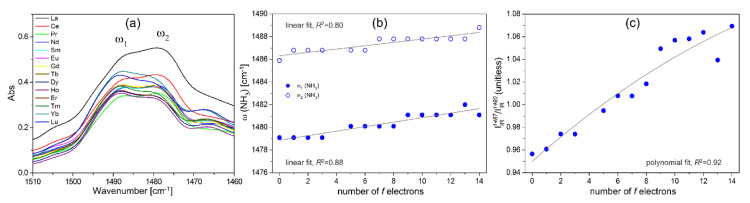
Juxtaposition of the IR spectral patterns for two *ω*(NH_3_) umbrella-like vibration bands of the Ln complexes with l-alanine (**a**). Correlations between the (**b**) IR band maxima and (**c**) IR intensity ratio of the *ω*(NH_3_) vibration bands at *ca.* 1487 and 1480 cm^−1^ and the number of the 4*f* electrons in the Ln atoms.

Often, the VCD bands directly connected to the chiral group are quite intense and robust. The studied structures have two β(C*H) bending vibrations taking place in two different planes. One is positioned at *ca.* 1345 and the other at 1300 cm^−1^ and they both exhibit regular band position and intensity ratio trends with the number of 4*f* electrons (Figure 10). Notice, that the lanthanide contraction effect on IR intensity is observed in Figure 10c and that although the correlation with the VCD intensity ratio is weak (Figure 10d), a suggestion that it can be ever observed has not have been given, so far. Quite unexpectedly, the vibrations of the moiety positioned far from the central ion, such as the ω(NH_3_) umbrella vibrations positioned at *ca.* 1487 and 1480 cm^−1^, also correlate with the number of 4*f* electrons (Figure 11). Both, the positions and intensities of these two IR bands linearly correlate with the number of 4*f* electrons: The IR intensity of the band at *ca.* 1487 cm^−1^ increases while that at *ca.* 1480 cm^−1^ decreases. However, the VCD baseline variation makes obtaining a strong correlation quite unlikely.

### 2.4. Simulated Molecular Structure and Vibrational Spectra

**Figure 12:**
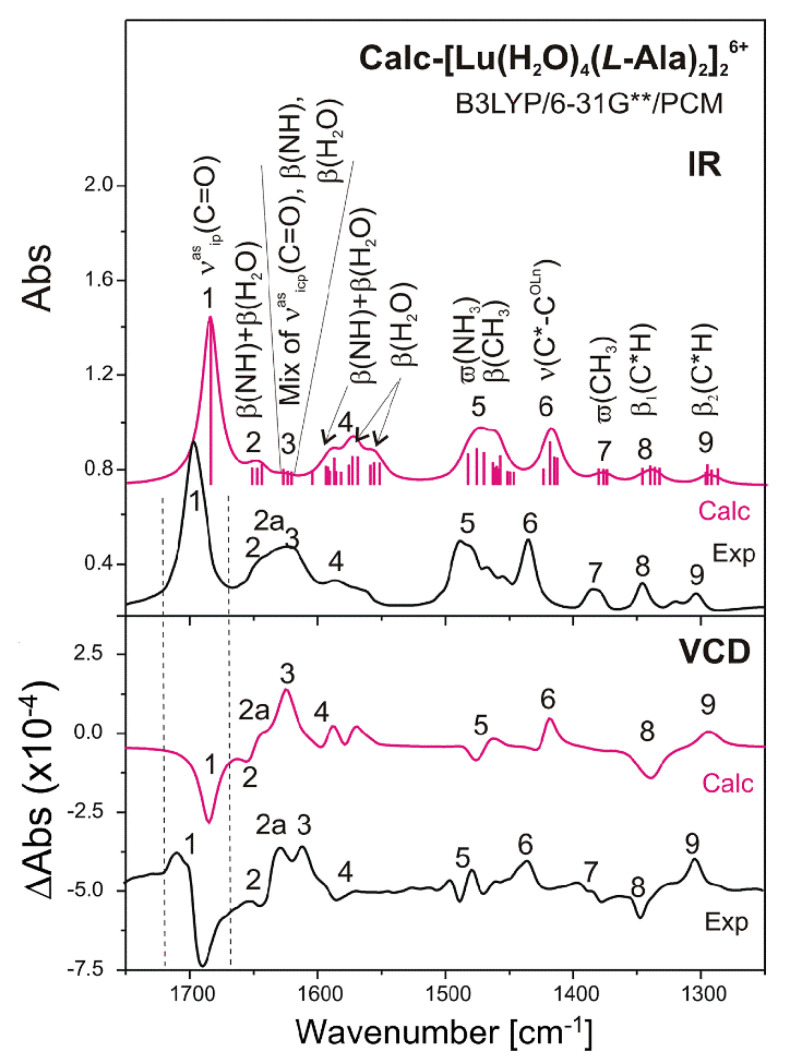
Assignment of IR and VCD bands of the [Lu(H_2_O)_4_(L-Ala)_2_]_2_(ClO_4_)_6_ complex in the solid state based on the simulated spectra of the [Lu(H_2_O)_4_(L-Ala)_2_]_2_^6+^ cation calculated with the B3LYP functional, 6-31G**(C,O,N,H), and SDD(Lu) basis set and pseudopotential plus PCM(water) solvation model. The calculated spectra are shifted by 50 cm^−1^ towards lower wavenumbers.

Computations of open shell systems are still challenging [78] and among the studied complexes, only the La^3+^ and Lu^3+^ ones have a closed shell configuration, but in addition only Lu^3+^ has fully filled *f*-orbitals. This is why the Lu^3+^ dimeric complex is the most realistic model of the complex dimers studied. Thus, for this system various simplifications, approaches and computational levels were tested to obtain the best agreement with the experimental geometrical and spectral data. First, in the calculations the ClO_4_^−^ counter ions were omitted due to convergence problems. Thus, charged (6+) systems were considered instead of the neutral ones observed in crystals. Secondly, to mimic the bulk crystal structure or solvent, and to prevent the dissociation or strong rearrangement of coordinated water molecules in the metal sphere the implicit Polarizable Continuum Model (PCM) [79,80] was applied. Without the PCM constraints, strong geometric deformations occurred. In contrary, the use of the PCM approach led to a better agreement between the computational and experimental X-ray data (Table S9). As a consequence, the experimental solid-state IR and VCD spectra were better reproduced as well (Figure S6). On the other hand, we found no unique indications as to which basis set is better to use. Two 6-31G** and TZVP basis sets were tested for all atoms but Lu and some geometrical parameters were better reproduced by the former while others by the latter basis set (Table S9). However, it seems that the B3LYP/6-31G**/PCM level is the best for reproduction of the experimental IR and VCD spectra (Figure 12 and Figure S6). Nevertheless, for the Lu system a noticeable discrepancy occurs in the 1675−1550 cm^−1^ range and there are two possible reasons for this disagreement. Firstly, the ν(C=O) VCD range is challenging for both the experiment and calculations as the band is seldom strong and predicting its rotatory strength may strongly depend on the computational level applied, the inclusion of a solvent effect or even the grid step [72,76]. Secondly, it is difficult to choose the model to reproduce the infinite periodic structure. Although some algorithms exist for calculating the IR spectra of periodic systems, they are still not available for the solid-state VCD spectra. Thirdly, considering the charged instead of neutral systems introduces some discrepancies between the calculated and experimental data. In our previous studies dealing with the VCD solid-state spectra of the H-bonded chain systems [76] we demonstrated that to satisfactory reproduce experimental data one has to consider a crystal fragment rather than not a single molecule. Here, the crystal packing is stabilized by the N−H···O_(ClO4)_, O−H_(H2O)_···O_(ClO4)_, and O−H_(H2O)_···O_(H2O)_ intermolecular interactions. Unfortunately, it was not possible to consider such interactions by a simple extension of the crystal structure due to large memory and computer time requirements for such systems.

Despite some discrepancy between the experimental and calculated spectra of the Lu system, it is possible to make the bands assignment somehow universal for the studied type of structures (Figure 12). However, the overall understanding of the VCD spectra is still limited and the spectral patterns presented in Figure 12 require comments. First, the ν_ip_(C=O) VCD (in-phase) bands demonstrate the +/− pattern while at the calculated spectra only one down-directed band is predicted. We think that this dissimilarity is a result of the difference between the species observed experimentally and those considered in the calculations. In the former, two lanthanide moieties in non-centrosymmetric arrangements occur whereas in calculations the Lu complexes have converged to a much more symmetric system than in reality. This is why in the experimental spectra two types of ν_ip_(C=O) vibrations are observed: weaker, symmetric (ν^s^_ip_(C=O)), up-directed and located at *ca.* 1725 cm^−1^; and stronger, antisymmetric (ν^as^_ip_(C=O)), down-directed and located at *ca.* 1675 cm^−1^. In calculations only the latter band is seen because the intensity of the former one is too weak. The experimental β(NH) and β(H_2_O) bands with −/+ VCD pattern at *ca.* 1650 cm^−1^ are fairly reproduced by the calculations, but the up-directed β(H_2_O) band at 1625 cm^−1^ is much stronger in the experimental image (Figure 12). The ν^as^_icp_(C=O) in-counter-phase VCD band at *ca.* 1650 cm^−1^ is up-directed in both experimental and computational spectra (Figure 12). Then, a weak contour of coupled bending NH and H_2_O vibrations at *ca.* 1575 cm^−1^ is correctly predicted. In the 1500−1200 cm^−1^ range of the VCD spectra, there are several bands (CH bend and deformation, NH deformation, and C−C stretching) which are quite well reproduced by the calculations (Figure 12).

The B3LYP/6-31G**(C,O,N,H)/SDD+PP(Ln)/PCM(water) level was used for the calculations of the all remaining lanthanides complexes for which all possible electron configurations were considered. The electron configurations of single lanthanide Ln^3+^ ions are inappropriate because the systems studied here have two Ln^3+^ ions in the elementary unit. Thus, to establish multiplicity of (Ln^3+^)_2_, the number of *f*-electrons accounting for multiplicity had to be doubled: For example, for (Nd^3+^)_2_ six *f*-electrons we had to consider all multiplicities from septet (all unpaired *f*-electrons) to singlet state (all paired *f*-electrons). The optimization of the open-shell systems was very onerous and it quite often failed at the convergence step. It was especially difficult for the systems with a high number of possible electronic configurations: e.g., for Tb dimeric structure—for each possible multiplicity the convergence failed sometimes without even one optimization step after two weeks of calculations by multiprocessor computations in a supercomputer center. This denotes that for different multiplicities the starting geometry is hardly predictable and requires finding the starting point in a hit-and-trial “method”. For the Dy system, the singlet, triplet, and nonet structures were obtained, while optimization of the quintet and septet structures failed. Moreover, for many systems, some low imaginary frequencies connected with water liberation modes occurred. Avoiding them would require further laborious calculations involving restarting the system from only slightly modified geometries. Nevertheless, based only on the correctly converged systems we can state that the higher the multiplicity the lower the energy. Moreover, the energy differences between the systems of increasing multiplicity are high indeed (Table S10). Therefore, it is most probable that we deal with very high-spin systems. Additionally, both the IR and VCD spectra are strongly electron-configuration-dependent. To shed some light on the multiplicity problem in the context of the calculated spectra we demonstrate some results obtained for the Yb and Nd systems.

**Figure 13:**
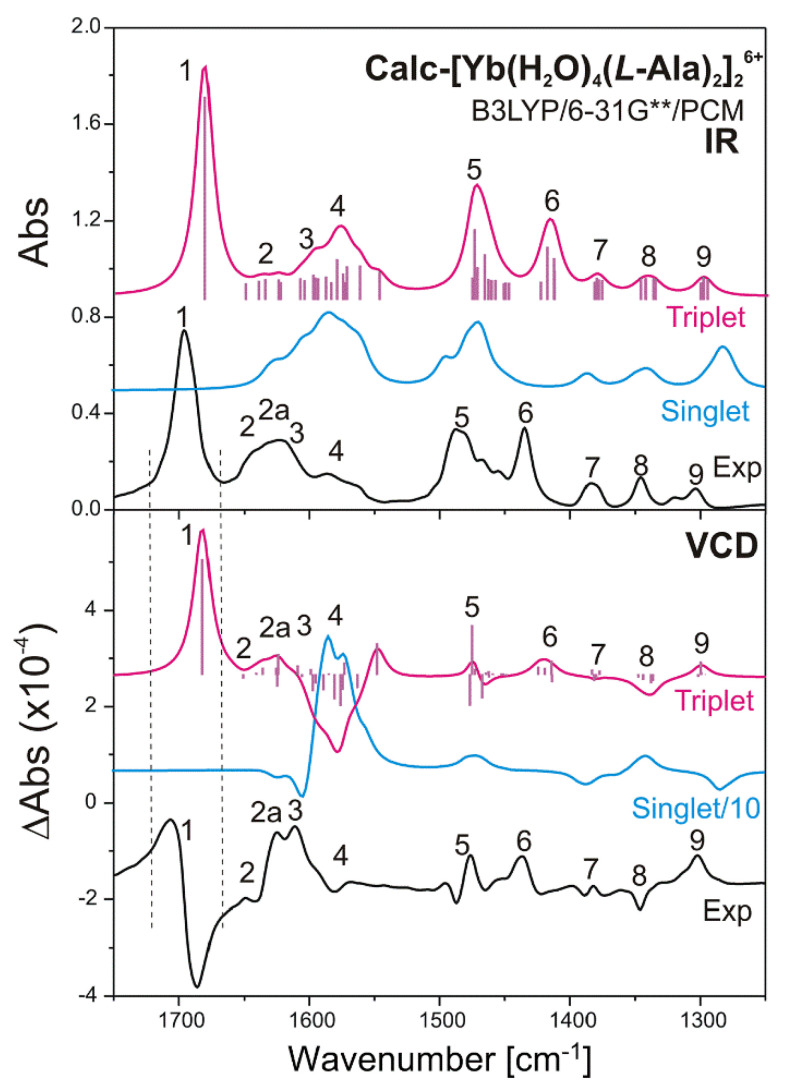
Comparison of the experimental solid-state IR and VCD spectra of [Yb(H_2_O)_4_(L-Ala)_2_]_2_(ClO_4_)_6_ with the calculated ones for singlet and triplet states of [Yb(H_2_O)_4_(L-Ala)_2_]_2_^6+^ with the B3LYP functional, 6-31G**(C,O,N,H) and SDD(Yb) basis set and pseudopotential plus PCM(water) solvation model. The calculated spectra are shifted by 50 cm^−1^ towards lower wavenumbers.

For the Yb complex, only the singlet and triplet states exist. According to the calculations, the triplet is more stable than the singlet by more than 80 kcal/mol (Table S10). The geometry calculated for the triplet is in better agreement with the experimental values than for the singlet, except for the sole interatomic Yb···Yb distance which is significantly underestimated (Table S11). Use of triplet geometry also gives a better agreement between the experimental and computational IR spectra (Figure 13). Based on the singlet state geometry, even the separation of the v(C=O) band is not predicted. Instead, the band is shifted towards lower wavenumbers and it overlaps with the other bands present in the 1650−1550 cm^−1^ range. However, even for the triplet state structure, the concordance between the experimental and computational VCD spectra in the 1750−1550 cm^−1^ range is not satisfactory. Nevertheless, below 1550 cm^−1^, the agreement is much better and the bands from 5th to 9th are quite well reproduced (Figure 13). Very surprisingly, below 1550 cm^−1^, the singlet and triplet VCD spectra of the Yb system are almost mutual mirror images. Thus, the calculated singlet state geometry is definitely inadequate for reproducing the IR/VCD experimental data in the whole spectral range.

The Nd structure septet is predicted to be the most stable and to be lower than the energy of the singlet, triplet, and quintet state by 180, 26, and 20 kcal/mol, respectively (Table S10). It is interesting that only the septet and quintet state geometries fit the X-ray geometry (determined at 100 K) of the lightest lanthanides with two alanine bridging modes: *µ*_2_-O,O’ and *µ*_2_-O,O,O’ (type **II**, Table S12). Significantly, the geometry of the moieties further away from the metal centers is ion-multiplicity-insensitive. The calculated IR and VCD spectra of the Nd dimer strongly depend on the electron configuration of the system (Figure S7). Indeed, as for the Yb complex in the singlet state, a separated v(C=O) band is not predicted by the IR spectrum of the singlet Nd structure. The IR spectrum of triplet is in the best agreement with the experimental one, however, this is not the case for the VCD spectrum. Although, the IR spectra of quintet and septet are identical, the VCD spectra differ in the 1650−1550 cm^−1^ range. In general, for the VCD spectra the agreement between experimental and computational spectra is low. However, this discrepancy may be due to the fact that the spectra were measured at room temperature at which the complex has a slightly different geometry than the geometry obtained in the calculations at 0 K (Figure 2).

## 3. Materials and Methods

All chemicals used were purchased from commercially available sources. The IR and VCD spectra of both enantiomers of alanine and their lanthanide(III) complexes were measured in the range 2000–1200 cm^−1^ at a resolution of 4 cm^−1^ on a Jasco FVS-6000 VCD spectrometer (Tokyo, Japan) equipped with the single PEM. The KBr pellets were prepared by mixing the studied compound with KBr matrix in mortar (*ca*. 1 and 100 mg, respectively) and pressed with *ca.* 9T for 3 min. The sample weights were adjusted to achieve IR absorbance between 0.7–0.9 for the most intensive ν(C=O) band. To average the polarizations of crystallites during the measurements the pellets were rotated with medium speed (2 rotations/min) using a special holder. To improve the signal-to-noise ratio, 10,000 scans were recorded and every measurement took approximately 2 h.

### 3.1. Preparation of the Complexes

All complexes were prepared in the same way using the following procedure: Ln_2_O_3_ (0.300 g) (exceptions: Tb_4_O_7_ (0.150 g) and Pr_6_O_11_ (0.150 g)) was dissolved under stirring with or without heating in a mixture containing concentrated aqueous solution of perchloric acid (0.5 mL) and deionized water (0.5 mL). After obtaining a clear solution of the lanthanide(III) perchlorate the solvent and the excess of acid were evaporated until a visible white fume was obtained. In the case of cerium its perchlorate salt (Ce(ClO_4_)_3_·6H_2_O (0.500 g)) was used directly. The resulting solid lanthanide(III) perchlorate was dissolved in 1.0 mL of deionized water and the requisite amount of l- or d-alanine was added to maintain the molar ratio of Ln to alanine as 1 to 2 (for Nd–Lu) or 1 to 1 (La–Pr). The pH of the prepared mixture was adjusted to a value of 3.5–4.0 by the addition of a concentrated NaOH solution. During the stirring and heating of the solution for a few hours, its volume was reduced until crystals began to precipitate. Then, the whole was left at room temperature. The crystalline material was isolated from the solution and dried under vacuum in a desiccator before IR and VCD measurements. The complexes were obtained in moderate yields as follows for La—0.390 g (62%), Ce—0.205 g (65%), Pr—0.070 g (69%), Nd—0.850 g (69%), Sm—0.720 g (60%), Eu—0.525 g (45%), Gd—0.480 g (41%), Tb—0.124 g (44%), Dy—0.540 g (47%), Ho—0.623 g (55%), Er—0.600 g (53%), Tm—0.470 g (42%), Yb—0.570 g (52%) and Lu—0.490 g (45%) (data presented for the Ln complexes with l-alanine).

### 3.2. Single-Crystal X-ray Diffraction

The diffraction data of suitable single crystals of studied lanthanide(III) complexes were collected on a Rigaku SuperNova (dual source) four circle diffractometer equipped with an Eos CCD detector (Oxford, UK). The measurements were performed using a mirror-monochromated Mo Kα radiation (λ = 0.71073 Å) from a microfocus Mova X-ray source. All the required procedures including data collection, data reduction and multi-scan absorption correction were performed using CrysAlis PRO software (version 1.171.38.41, Oxford, UK). The structures were solved by direct methods and refined by the full matrix least-squares technique on F^2^ data. All non-hydrogen atoms, with only a few exceptions for disordered perchlorate ions, were refined anisotropically while the hydrogen atoms were inserted in calculated positions or in those from the Fourier matrix and refined isotropically using standard parameters. All calculations were performed with the use of the OLEX2 crystallographic software [81] equipped with SHELX programs [82]. A MERCURY program [83] was applied for a graphical representation of the crystal structures. Selected crystallographic parameters and refinement details for the l-alanine complexes obtained from measurements at 100 K are listed in Table 1 and Table 2, while for the related d-alanine compounds in Tables S1 and S2. Additional crystallographic data obtained from measurements at ambient temperature (292 K) are collected in Table S6.

### 3.3. DFT Calculations

To interpret the experimental solid-state IR and VCD spectra of the chiral Pr, Nd, Tb, Dy, Ho, Er, Tm, Yb, and Lu complexes, we simulated their model structures by using quantum-chemical methods. Because the open-shell calculations of the 4*f*-systems are very challenging for the single-reference *ab initio* calculations often the La surrogate-based complexes, without the *f*-electrons present in La, are applied to interpret the chiroptical spectra of the other Ln structures [56]. In the case of dimeric Ln systems, reproducing the spectra is even more demanding as their multiplicity is unknown. Therefore, it was necessary to consider as many probable multiplicities as possible. In consequence, in the extreme Tb complex case, it was inevitable to consider as many as seven electron configurations: From singlet to tridecet. The initial geometry of the studied systems was based on their crystal geometries from which six ClO_4_^−^ counterions were removed. Therefore, the structures were set to have a 6+ charge. Moreover, eight water molecules in the metal coordination sphere were considered. The optimization of some systems failed as some water molecules dissociated. Therefore, to mimic the bulk solvent and to prevent the escape of water from the metal sphere, the implicit Polarizable Continuum Model (PCM) [79,80] was applied. The studied systems were optimized by using the hybrid Becke three parameter Lee-Yang-Parr, B3LYP [84,85], DFT functional, the SDD pseudopotential and basis set for Ln [86,87], and the 6-31G** basis set [88] for the other atoms. In the case of Lu, the TZVP [89] basis set was also applied. Shapes of the IR and VCD spectra were simulated using the Lorentzian functions with the 8 cm^−1^ peak half-width at half maximum. All calculations were performed by using the Gaussian 09 program [90]. The Cartesian Coordinates of the discussed complexes are gathered in Table S13.

## 4. Conclusions and Summary

Detailed structural studies on dimeric lanthanide complexes with both alanine enantiomers revealed that two types of dimers (**I** and **II**) were formed depending on the lanthanide atom and the temperature. In all complexes the metal ion is coordinated by four water molecules and four alanine groups form bridges between the two [Ln(H_2_O)_4_]^3+^ parts. Four alanine molecules in dimer **I** form the *μ*_2_-O,O’- bridges, while besides two such bridges in the dimer **II**, two *μ*_2_-O,O,O’-connections also exist. The dimer **I** was observed for heavy lanthanide complexes at 100 K. A similar structure was also found for all lanthanides at room temperature. Dimer **II** was found only for the light lanthanides (La–Nd) when measured at 100 K. It was proved that with a significant decrease in temperature (from 292 to 100 K) their crystals undergo an isostructural phase transition transforming two *μ*_2_-O,O’- bridges into *μ*_2_-O,O,O’- ones.

Despite the fact that at room temperature all studied complexes exhibit similar structures a deeper insight into the IR and VCD spectra reveals some correlations or trends between spectral features and the d(Ln–O1) distances. The ν(C=O), ν(C*−C^OLn^), ω(NH_3_), and β(C*–H) IR band positions and intensities vary with the lanthanides regardless of the distance of a vibrating moiety from the metal center. Unexpectedly, the ν(C=O) VCD bands, which tend to be small or inactive in solutions, are quite intense and exhibit spectral patterns which vary with the lanthanide. Several IR frequencies reveal a significant lanthanide contraction effect and were well correlated with the number of 4*f* electrons in the Ln-central ion. Moreover, some VCD band positions weakly correlate with Ln, while the VCD intensities show at most some trends. This is the first example of the lanthanide contraction effect observed in the VCD spectra for frequencies and strong suggestion that the effect can also be observed for the VCD intensities. The VCD spectra are 4-orders weaker than the IR ones, and the VCD baseline often varies. This is why finding correlations for the VCD spectral features is indeed difficult. Also, this is why for two enantiomers, it is difficult to obtain a fair mirror-image. However, for the heavier lanthanides (from Dy to Lu) the VCD display satisfactory mirror-image patterns, while for some lighter ones the patterns are much more erratic.

According to the calculations, the higher the multiplicity of the Ln atom the higher the stability of the system. The molecular geometry observed in crystals at low temperature is properly predicted based on the highest-spin structures. Indeed, the calculated highest-spin Yb and Nd complexes match the experimental geometry the best. In the triplet Yb state, the alanine ligands in the calculated complex form only one, *µ*_2_-O,O’- bridge (type **I**) while in the quintet and septet states of Nd, two alanine bridging modes, *µ*_2_-O,O’- and *µ*_2_-O,O,O’-, are predicted (type **II**). The IR and VCD spectra strongly depend on the Ln electron configuration but the best overall agreement was reached based on the Lu complex, which is the only system with a fully filled *f*-shell.

Strikingly, as in the structures described in this study, in biomacromolecules the lanthanide cations are often located between the symmetry equivalent molecules, where they tend to form polynuclear complexes [20]. This is why, the observed *μ*_2_-O,O’- and *μ*_2_-O,O,O’ alanine bridges between metal centers are fair model structures for lanthanide interactions in a bioenvironment. Noticeably, the transformation between *μ*_2_-O,O’- and *μ*_2_-O,O,O’- bridges in transition between dimers **I** and **II** suggests that similar transformation can occur in biosystems. Moreover, our IR and VCD spectroscopy findings reveal that despite the similarity between the lanthanide ion’s physical and chemical properties, the differences are clear and spectroscopically detectable. They can be used in monitoring the lanthanide-biomolecule interactions.

## Figures and Tables

**Table 1:** Crystal data and structure refinement details obtained from measurements at 100 K for studied l-alanine complexes with light lanthanides.

Ln	La	Ce	Pr	Nd
Chemical formula	C_12_H_44_Cl_6_N_4_O_40_La_2_	C_12_H_44_Cl_6_N_4_O_40_Ce_2_	C_12_H_44_Cl_6_N_4_O_40_Pr_2_	C_12_H_44_Cl_6_N_4_O_40_Nd_2_
Formula weight	1375.03	1377.45	1379.03	1385.69
*λ* (Mo Kα) (Å)	0.71073	0.71073	0.71073	0.71073
Crystal system	triclinic	triclinic	triclinic	triclinic
Space group	*P* 1	*P* 1	*P* 1	*P* 1
*a* (Å)	10.6638(4)	10.61089(15)	10.5830(2)	10.5197(3)
*b* (Å)	11.1982(4)	11.21068(13)	11.1985(2)	11.1630(4)
*c* (Å)	11.3160(5)	11.26955(18)	11.2455(3)	11.1905(4)
α (°)	79.400(3)	79.5635(12)	79.7557(18)	79.937(3)
β (°)	67.118(4)	67.1951(15)	67.394(2)	67.541(3)
*γ* (°)	65.265(3)	65.3845(13)	65.4262(19)	65.572(3)
*V* (Å^3^)	1130.38(8)	1123.17(3)	1118.66(4)	1105.50(7)
*Z*	1	1	1	1
*D*_calc._ (g·cm^−3^)	2.020	2.036	2.047	2.081
*µ* (mm^−1^)	2.337	2.476	2.629	2.805
*F*(000)	680	682	684	686
Crystal size (mm)	0.15 × 0.12 × 0.04	0.18 × 0.16 × 0.10	0.28 × 0.28 × 0.15	0.20 × 0.18 × 0.10
Reflections collected	24,374	101,937	62,860	25,043
Unique reflections	12,027	12,542	12,497	12,314
Reflections *I* > 2*σ*(*I*)	11,309	12,238	12,118	11,798
*R*_int_	0.0288	0.0326	0.0387	0.0250
Restraints/parameters	4/638	3/626	3/614	14/635
Goodness-of-fit	1.051	1.050	1.030	1.030
*R*_1_, *wR*_2_ (*I* > 2*σ*(*I*))	0.0335, 0.0688	0.0200, 0.0464	0.0248, 0.0582	0.0243, 0.0530
*R*_1_, *wR*_2_ (all data)	0.0369, 0.0712	0.0209, 0.0470	0.0261, 0.0593	0.0264, 0.0546
Peak/hole (e·Å^−3^)	2.146/−1.970	1.506/−0.797	0.992/−0.683	0.619/−0.888

**Table 2:** Crystal data and structure refinement details obtained from measurements at 100 K for studied l-alanine complexes with heavy lanthanides.

**Ln**	**Tb**	**Dy**	**Ho**	**Er**
Chemical formula	C_12_H_44_Cl_6_N_4_O_40_Tb_2_	C_12_H_44_Cl_6_N_4_O_40_Dy_2_	C_12_H_44_Cl_6_N_4_O_40_Ho_2_	C_12_H_44_Cl_6_N_4_O_40_Er_2_
Formula weight	1415.05	1422.21	1427.07	1431.73
*λ* (Mo Kα) (Å)	0.71073	0.71073	0.71073	0.71073
Crystal system	triclinic	triclinic	triclinic	triclinic
Space group	*P* 1	*P* 1	*P* 1	*P* 1
*a* (Å)	10.7620(3)	10.7445(4)	10.7253(3)	10.72306(14)
*b* (Å)	10.8068(2)	10.7596(4)	10.7486(3)	10.75190(16)
*c* (Å)	11.3278(2)	11.3154(4)	11.3110(3)	11.29447(17)
α (°)	79.2425(18)	79.191(3)	79.288(2)	79.3033(13)
β (°)	65.411(2)	65.306(3)	65.354(3)	65.4293(14)
*γ* (°)	67.538(2)	67.622(3)	67.705(3)	67.6274(13)
*V* (Å^3^)	1106.51(4)	1098.38(7)	1095.98(6)	1094.54(3)
*Z*	1	1	1	1
*D*_calc._ (g·cm^−3^)	2.124	2.150	2.162	2.172
*µ* (mm^−1^)	3.652	3.861	4.071	4.295
*F*(000)	696	698	700	702
Crystal size (mm)	0.12 × 0.10 × 0.04	0.12 × 0.10 × 0.06	0.14 × 0.12 × 0.05	0.18 × 0.15 × 0.06
Reflections collected	30,268	38,791	24,694	93,208
Unique reflections	11,792	11,710	12,191	13,987
Reflections *I* > 2*σ*(*I*)	11,149	11,305	11,583	13,531
*R*_int_	0.0355	0.0302	0.0285	0.0375
Restraints/parameters	3/612	5/612	17/642	5/618
Goodness-of-fit	1.054	1.040	1.045	1.045
*R*_1_, *wR*_2_ (*I* > 2*σ*(*I*))	0.0249, 0.0522	0.0212, 0.0463	0.0251, 0.0494	0.0205, 0.0222
*R*_1_, *wR*_2_ (all data)	0.0277, 0.0540	0.0225, 0.0471	0.0277, 0.0512	0.0457, 0.0468
Peak/hole (e·Å^−3^)	0.924/−0.780	0.809/−0.678	0.910/−0.682	0.939/−0.789
**Ln**	**Tm**	**Yb**	**Lu**	
Chemical formula	C_12_H_44_Cl_6_N_4_O_40_Tm_2_	C_12_H_44_Cl_6_N_4_O_40_Yb_2_	C_12_H_44_Cl_6_N_4_O_40_Lu_2_	
Formula weight	1435.07	1443.29	1447.15	
*λ* (Mo Kα) (Å)	0.71073	0.71073	0.71073	
Crystal system	triclinic	triclinic	triclinic	
Space group	*P* 1	*P* 1	*P* 1	
*a* (Å)	10.7174(3)	10.7127(2)	10.7019(3)	
*b* (Å)	10.7660(4)	10.73533(18)	10.7326(3)	
*c* (Å)	11.2977(4)	11.2818(2)	11.2736(3)	
α (°)	79.468(3)	79.3635(16)	79.370(2)	
β (°)	65.476(3)	65.397(2)	65.470(3)	
*γ* (°)	67.710(3)	67.7669(18)	67.668(3)	
*V* (Å^3^)	1096.85(7)	1091.39(4)	1089.10(6)	
*Z*	1	1	1	
*D*_calc._ (g·cm^−3^)	2.173	2.196	2.206	
*µ* (mm^−1^)	4.505	4.747	4.996	
*F*(000)	704	706	708	
Crystal size (mm)	0.12 × 0.10 × 0.04	0.18 × 0.10 × 0.08	0.20 × 0.18 × 0.05	
Reflections collected	22,981	39,938	24,285	
Unique reflections	11,661	11,636	12,029	
Reflections *I* > 2*σ*(*I*)	11,086	11,201	11,280	
*R*_int_	0.0270	0.0306	0.0341	
Restraints/parameters	6/588	6/606	4/581	
Goodness-of-fit	1.026	1.047	1.018	
*R*_1_, *wR*_2_ (*I* > 2*σ*(*I*))	0.0258, 0.0524	0.0199, 0.0429	0.0281, 0.0570	
*R*_1_, *wR*_2_ (all data)	0.0281, 0.0537	0.0215, 0.0436	0.0317, 0.0593	
Peak/hole (e·Å^−3^)	0.639/−0.679	0.619/−0.645	0.937/−1.123	

## Supplementary Materials

CCDC 2000724-2000752 contain the supplementary crystallographic data for this paper. These data can be obtained free of charge from the CCDC via http://www.ccdc.cam.ac.uk/data_request/cif. The other Supplementary Materials are available online, Figure S1. Molecular structures of cationic dimeric complexes [Ln(H_2_O)_4_(L-Ala)_2_]_2_^6+^ obtained from low temperature (100 K) measurements. Displacement ellipsoids are drawn at the 50% probability level. Perchlorate anions are omitted for clarity. Figure S2. Graphical distribution of bond lengths and distances obtained from measurements at 100 K for both lanthanide atoms in dimeric complexes [Ln(H_2_O)_4_(D-Ala)_2_]_2_(ClO_4_)_6_. Figure S3. Graphical distribution of bond lengths and distances obtained from measurements at 292 K for both lanthanide atoms in dimeric complexes [Ln(H_2_O)_4_(L-Ala)_2_]_2_(ClO_4_)_6_. Figure S4. Juxtaposition of the experimental solid-state VCD spectra of L-Ala (black) and D-Ala (red) dimeric lanthanide complexes with their IR counterparts (green) in the ν(C=O) vibrational range (measured in KBr pellets). Figure S5. Variation of the VCD intensity of the ν^1^(C=O) and ν^2^(C=O) VCD bands with the number of 4*f* electrons in the Ln complexes with l- (circles) and d-alanine (triangles). Figure S6. Comparison of the experimental solid-state IR and VCD spectra of [Lu(H_2_O)_4_(L-Ala)_2_]_2_(ClO_4_)_6_ with the calculated ones obtained for singlet state of [Lu(H_2_O)_4_(L-Ala)_2_]_2_^6+^ with the B3LYP functional, different basis sets for C,O,N,H atoms, SDD basis sets and pseudopotential for Lu, and presence or absence of the PCM(water) solvation model. The calculated spectra are shifted by 50 cm^−1^ towards lower wavenumbers. Figure S7. Comparison of the experimental solid-state IR and VCD spectra of [Nd(H_2_O)_4_(L-Ala)_2_]_2_(ClO_4_)_6_ with the calculated ones for different multiplicity states of [Nd(H_2_O)_4_(L-Ala)_2_]_2_^6+^ with the B3LYP functional, 6-31G**(C,O,N,H) and SDD(Nd) basis set and pseudopotential plus PCM(water) solvation model. The calculated spectra are shifted by 50 cm^−1^ towards lower wavenumbers. Table S1. Crystal data and structure refinement details for studied d-alanine complexes with light lanthanides obtained from measurements at 100 K. Table S2. Crystal data and structure refinement details for studied d-alanine complexes with heavy lanthanides obtained from measurements at 100 K. Table S3. Crystal data for studied l-alanine complexes with Sm, Eu and Gd obtained from measurements at 100 K. Table S4. Selected bond lengths and distances [Å] for dimeric lanthanide complexes with l-alanine obtained from measurements at 100 K. Table S5. Selected bond lengths and distances [Å] for dimeric lanthanide complexes with d-alanine obtained from measurements at 100 K. Table S6. Crystal data and structure refinement details for studied l-alanine complexes with light lanthanides obtained from measurements at 292 K. Table S7. Comparison of bond lengths and distances [Å] for dimeric l-alanine complexes with light lanthanides obtained from crystal structure measurements carried out at 100 and 292 K. The highest differences in distances between both measurements for the respective lanthanide are marked in red. Table S8. Selected bond lengths and distances [Å] for dimeric lanthanide complexes with l-alanine obtained from measurements at 292 K. Table S9. Comparison of distances (Å) in [Lu(H_2_O)_4_(L-Ala)_2_]_2_(ClO_4_)_6_ and [Lu(H_2_O)_4_(L-Ala)_2_]_2_^6+^ systems obtained with X-ray measurements and calculations at different computational levels, respectively. The best agreement with the experimental data is indicated in grey. Table S10. The total (E, au) and relative (ΔE, kcal/mol) energies referred to the most stable form for different multiplicity states of the [Ln(H_2_O)_4_(L-Ala)_2_]_2_^6+^ systems obtained at the B3LYP/6-31G**(C,N,O,H)/SDD(Ln)+PP(Ln)/PCM(H_2_O) level. M stands for multiplicity (M1–singlet, M3–triplet, M5 – quintet *etc*.), nc – not converged. Table S11. Comparison of distances (Å) in [Yb(H_2_O)_4_(L-Ala)_2_]_2_(ClO_4_)_6_ and [Yb(H_2_O)_4_(L-Ala)_2_]_2_^6+^ systems obtained respectively with X-ray measurements and calculations performed for singlet ant triplet states at the B3LYP/6-31G**(C,O,N,H)/SDD(Yb)+PP(Yb)/PCM(water) level. The best agreement with the experimental data is indicated in grey. Table S12. Comparison of distances (Å) in [Nd(H_2_O)_4_(L-Ala)_2_]_2_(ClO_4_)_6_ and [Nd(H_2_O)_4_(L-Ala)_2_]_2_^6+^ systems obtained respectively with X-ray measurements and calculations performed for different multiplicity states at the B3LYP/6-31G**(C,O,N,H)/SDD(Nd)+PP(Nd)/PCM(water) level. The best agreement with the experimental data is indicated in grey. Table S13. Cartesian Coordinates of the [Ln(H_2_O)_4_(L-Ala)_2_]_2_^6+^ complexes calculated at different computational levels (B3LYP/SDD(Ln)+PP(Ln)).
