# Supplementary material for: Chiral Lanthanide Complexes with l- and d-Alanine: An X-ray and Vibrational Circular Dichroism Study
Source: Molecules. 2020 Jun 12;25(12):2729. doi: 10.3390/molecules25122729 (PMC7357152; doi:10.3390/molecules25122729)
Supplement: Supplementary file 1 [file molecules-25-02729-s001.pdf]

molecules

Supplementary Materials

Chiral Lanthanide Complexes with L- and D-Alanine: An X-ray and Vibrational Circular Dichroism Study

Krzysztof Lyczko *, Joanna E. Rode and Jan Cz. Dobrowolski

Institute of Nuclear Chemistry and Technology, Dorodna 16, 03-195 Warsaw, Poland; J.Rode@ichtj.waw.pl (J.E.R.); j.dobrowolski@nil.gov.pl (J.C.D.)

* Correspondence: k.lyczko@ichtj.waw.pl

Contents

Table S1. Crystal data and structure refinement details for studied D-alanine complexes with light lanthanides obtained from measurements at 100 K.

Table S2. Crystal data and structure refinement details for studied D-alanine complexes with heavy lanthanides obtained from measurements at 100 K.

Table S3. Crystal data for studied L-alanine complexes with Sm, Eu and Gd obtained from measurements at 100 K.

Figure S1. Molecular structures of cationic dimeric complexes $[Ln(H_2O)_4(L\text{-}Ala)_2]_2^{6+}$ obtained from low temperature (100 K) measurements. Displacement ellipsoids are drawn at the 50% probability level. Perchlorate anions are omitted for clarity.

Table S4. Selected bond lengths and distances [Å] for dimeric lanthanide complexes with L-alanine obtained from measurements at 100 K.

Table S5. Selected bond lengths and distances [Å] for dimeric lanthanide complexes with D-alanine obtained from measurements at 100 K.

Figure S2. Graphical distribution of bond lengths and distances obtained from measurements at 100 K for both lanthanide atoms in dimeric complexes $[Ln(H_2O)_4(D\text{-}Ala)_2]_2(ClO_4)_6$.

Table S6. Crystal data and structure refinement details for studied L-alanine complexes with light lanthanides obtained from measurements at 292 K.

Table S7. Comparison of bond lengths and distances [Å] for dimeric L-alanine complexes with light lanthanides obtained from crystal structure measurements carried out at 100 and 292 K. The highest differences in distances between both measurements for the respective lanthanide are marked in red.

Table S8. Selected bond lengths and distances [Å] for dimeric lanthanide complexes with L-alanine obtained from measurements at 292 K.

Figure S3. Graphical distribution of bond lengths and distances obtained from measurements at 292 K for both lanthanide atoms in dimeric complexes $[Ln(H_2O)_4(L\text{-}Ala)_2]_2(ClO_4)_6$.

Figure S4. Juxtaposition of the experimental solid-state VCD spectra of L-Ala (black) and D-Ala (red) dimeric lanthanide complexes with their IR counterparts (green) in the ν(C=O) vibrational range (measured in KBr pellets).

Figure S5. Variation of the VCD intensity of the ν^1(C=O) and ν^2(C=O) VCD bands with the number of 4*f* electrons in the Ln complexes with L- (circles) and D-alanine (triangles).

Table S9. Comparison of distances (Å) in $[Lu(H_2O)_4(L\text{-}Ala)_2]_2(ClO_4)_6$ and $[Lu(H_2O)_4(L\text{-}Ala)_2]_2^{6+}$ systems obtained with X-ray measurements and calculations at different computational levels, respectively. The best agreement with the experimental data is indicated in grey.

Figure S6. Comparison of the experimental solid-state IR and VCD spectra of $[Lu(H_2O)_4(L\text{-}Ala)_2]_2(ClO_4)_6$ with the calculated ones obtained for singlet state of $[Lu(H_2O)_4(L\text{-}Ala)_2]_2^{6+}$ with the B3LYP functional, different basis sets for C,O,N,H atoms, SDD basis sets and pseudopotential for Lu, and presence or absence of the PCM(water) solvation model. The calculated spectra are shifted by 50 cm^{-1} towards lower wavenumbers.

Table S10. The total (E, au) and relative (ΔE, kcal/mol) energies referred to the most stable form for different multiplicity states of the $[Ln(H_2O)_4(L\text{-}Ala)_2]_2^{6+}$ systems obtained at the B3LYP/6-31G**(C,N,O,H)/SDD(Ln)+PP(Ln)/PCM(H_2O) level. M stands for multiplicity (M1–singlet, M3–triplet, M5 – quintet *etc*.), nc–not converged.

Table S11. Comparison of distances (Å) in $[Yb(H_2O)_4(L\text{-}Ala)_2]_2(ClO_4)_6$ and $[Yb(H_2O)_4(L\text{-}Ala)_2]_2^{6+}$ systems obtained respectively with X-ray measurements and calculations performed for singlet ant triplet states at the B3LYP/6-31G**(C,O,N,H)/SDD(Yb)+PP(Yb)/PCM(water) level. The best agreement with the experimental data is indicated in grey.

Table S12. Comparison of distances (Å) in $[Nd(H_2O)_4(L\text{-}Ala)_2]_2(ClO_4)_6$ and $[Nd(H_2O)_4(L\text{-}Ala)_2]_2^{6+}$ systems obtained respectively with X-ray measurements and calculations performed for different multiplicity states at the B3LYP/6-31G**(C,O,N,H)/SDD(Nd)+PP(Nd)/PCM(water) level. The best agreement with the experimental data is indicated in grey.

Figure S7. Comparison of the experimental solid-state IR and VCD spectra of $[Nd(H_2O)_4(L\text{-}Ala)_2]_2(ClO_4)_6$ with the calculated ones for different multiplicity states of $[Nd(H_2O)_4(L\text{-}Ala)_2]_2^{6+}$ with the B3LYP functional, 6-31G**(C,O,N,H) and SDD(Nd) basis set and pseudopotential plus PCM(water) solvation model. The calculated spectra are shifted by 50 cm^{-1} towards lower wavenumbers.

Table S13. Cartesian Coordinates of the $[Ln(H_2O)_4(L\text{-}Ala)_2]_2^{6+}$ complexes calculated at different computational levels (B3LYP/SDD(Ln)+PP(Ln)).

Table S1. Crystal data and structure refinement details for studied D-alanine complexes with light lanthanides obtained from measurements at 100 K.

**Ln**	**La**	**Ce**	**Pr**	**Nd**
Chemical formula	$C_{12}H_{44}Cl_6N_4O_{40}La_2$	$C_{12}H_{44}Cl_6N_4O_{40}Ce_2$	$C_{12}H_{44}Cl_6N_4O_{40}Pr_2$	$C_{12}H_{44}Cl_6N_4O_{40}Nd_2$
Formula weight	1375.03	1377.45	1379.03	1385.69
λ (Mo Kα) (Å)	0.71073	0.71073	0.71073	0.71073
Crystal system	triclinic	triclinic	triclinic	triclinic
Space group	P 1	P 1	P 1	P 1
a (Å)	10.6613(2)	10.61088(19)	10.5833(3)	10.54532(18)
b (Å)	11.1946(3)	11.2087(2)	11.1993(3)	11.18944(19)
c (Å)	11.3082(3)	11.27044(15)	11.2401(3)	11.20928(17)
α (°)	79.386(2)	79.6034(13)	79.739(2)	79.9554(14)
β (°)	67.146(2)	67.2103(14)	67.318(3)	67.5474(15)
γ (°)	65.218(2)	65.3642(17)	65.452(3)	65.5616(17)
V (Å^3)	1128.76(5)	1123.02(4)	1117.85(6)	1112.66(4)
Z	1	1	1	1
$D_{calc.}$ (g·cm^{-3})	2.023	2.037	2.049	2.068
μ (mm^{-1})	2.340	2.477	2.631	2.787
F(000)	680	682	684	686
Crystal size (mm)	0.30 x 0.18 x 0.04	0.15 x 0.12 x 0.08	0.12 x 0.10 x 0.06	0.28 x 0.20 x 0.08
Reflections collected	44216	78881	43304	56003
Unique reflections	10373	12541	12481	10706
Reflections $I > 2\sigma(I)$	9976	12062	11863	10408
R_{int}	0.0368	0.0343	0.0403	0.0361
Restraints/parameters	7 / 658	5 / 610	5 / 606	5 / 591
Goodness-of-fit	1.043	1.036	1.151	1.099
R_1, wR_2 ($I > 2\sigma(I)$)[a]	0.0294, 0.0666	0.0218, 0.0489	0.0331, 0.0804	0.0226, 0.0535
R_1, wR_2 (all data)[a]	0.0313, 0.0682	0.0236, 0.0498	0.0360, 0.0820	0.0239, 0.0543
Peak/hole (e·Å^{-3})	1.973/−1.133	1.378/−0.803	1.479/−1.288	0.724/−0.642

Table S2. Crystal data and structure refinement details for studied D-alanine complexes with heavy lanthanides obtained from measurements at 100 K.

**Ln**	**Tb**	**Dy**	**Ho**	**Er**	**Tm**	**Yb**	**Lu**
Chemical formula	$C_{12}H_{44}Cl_6N_4O_{40}Tb_2$	$C_{12}H_{44}Cl_6N_4O_{40}Dy_2$	$C_{12}H_{44}Cl_6N_4O_{40}Ho_2$	$C_{12}H_{44}Cl_6N_4O_{40}Er_2$	$C_{12}H_{44}Cl_6N_4O_{40}Tm_2$	$C_{12}H_{44}Cl_6N_4O_{40}Yb_2$	$C_{12}H_{44}Cl_6N_4O_{40}Lu_2$
Formula weight	1415.05	1422.21	1427.07	1431.73	1435.07	1443.29	1447.15
λ (Mo Kα) (Å)	0.71073	0.71073	0.71073	0.71073	0.71073	0.71073	0.71073
Crystal system	triclinic	triclinic	triclinic	triclinic	triclinic	triclinic	triclinic
Space group	*P* 1	*P* 1	*P* 1	*P* 1	*P* 1	*P* 1	*P* 1
a (Å)	10.76280(13)	10.74872(19)	10.7505(2)	10.74363(18)	10.7024(3)	10.71966(15)	10.71006(15)
b (Å)	10.80300(17)	10.7717(2)	10.7633(3)	10.76921(14)	10.7614(3)	10.74768(17)	10.73826(16)
c (Å)	11.34311(15)	11.3231(2)	11.3431(3)	11.32411(16)	11.2918(2)	11.28787(18)	11.27524(15)
α (°)	79.2649(12)	79.1835(16)	79.290(2)	79.2931(11)	79.3960(18)	79.3426(14)	79.3564(13)
β (°)	65.3835(12)	65.3352(18)	65.367(2)	65.3959(15)	65.498(2)	65.4408(15)	65.4537(13)
γ (°)	67.6430(13)	67.6105(17)	67.732(2)	67.6387(14)	67.757(2)	67.7145(15)	67.6453(13)
V (Å^3)	1108.27(3)	1100.95(4)	1103.49(5)	1101.08(3)	1094.78(5)	1093.91(3)	1090.35(3)
Z	1	1	1	1	1	1	1
$D_{calc.}$ (g·cm^{-3})	2.120	2.145	2.147	2.159	2.177	2.191	2.204
μ (mm^{-1})	3.646	3.852	4.043	4.270	4.513	4.736	4.990
F(000)	696	698	700	702	704	706	708
Crystal size (mm)	0.18 x 0.10 x 0.05	0.16 x 0.12 x 0.06	0.30 x 0.15 x 0.12	0.20 x 0.18 x 0.10	0.20 x 0.15x 0.08	0.15 x 0.10 x 0.07	0.18 x 0.12 x 0.05
Reflections collected	93880	71981	60184	95969	35800	58457	82933
Unique reflections	12368	11731	11771	11722	11667	11657	11625
Reflections $I > 2\sigma(I)$	11900	11275	11452	11325	11085	11232	11255
R_{int}	0.0385	0.0388	0.0347	0.0359	0.0336	0.0367	0.0406
Restraints/parameters	4 / 591	4 / 621	4 / 606	5 / 604	3 / 585	4 / 591	5 / 591
Goodness-of-fit	1.066	1.074	1.0585	1.030	1.023	1.043	1.041
R_1, wR_2 ($I > 2\sigma(I)$)[a]	0.0211, 0.0466	0.0234, 0.0536	0.0190, 0.0436	0.0217, 0.0233	0.0243, 0.0536	0.0219, 0.0496	0.01921, 0.0423
R_1, wR_2 (all data)[a]	0.0231, 0.0481	0.0253, 0.0549	0.0201, 0.0444	0.0511, 0.0524	0.0269, 0.0554	0.0236, 0.0508	0.0206, 0.0431
Peak/hole (e·Å^{-3})	0.966/−0.823	0.854/−0.766	0.810/−0.920	1.040/−0.653	0.982/−0.751	0.884/−0.674	0.907/−0.574

Table S3. Crystal data for studied L-alanine complexes with Sm, Eu and Gd obtained from measurements at 100 K.

Ln	Sm	Eu	Gd
Chemical formula	$C_{12}H_{44}Cl_6N_4O_{40}Sm_2$	$C_{12}H_{44}Cl_6N_4O_{40}Eu_2$	$C_{12}H_{44}Cl_6N_4O_{40}Gd_2$
Formula weight	1397.91	1401.13	1411.71
λ (Cu Kα) (Å)	1.54184	1.54184	1.54184
Crystal system	triclinic	triclinic	triclinic
Space group	*P* 1	*P* 1	*P* 1
a (Å)	11.2156(2)	11.2335(3)	11.2176(4)
b (Å)	20.2784(4)	20.2255(7)	20.2084(7)
c (Å)	25.7730(4)	25.7303(6)	25.7422(7)
α (°)	70.8492(17)	71.220(3)	71.502(3)
β (°)	89.5213(16)	89.814(2)	89.918(3)
γ (°)	89.6593(18)	89.643(3)	89.464(3)
V (Å^3)	5537.0(2)	5534.7(3)	5533.8(3)
Z	5	5	5

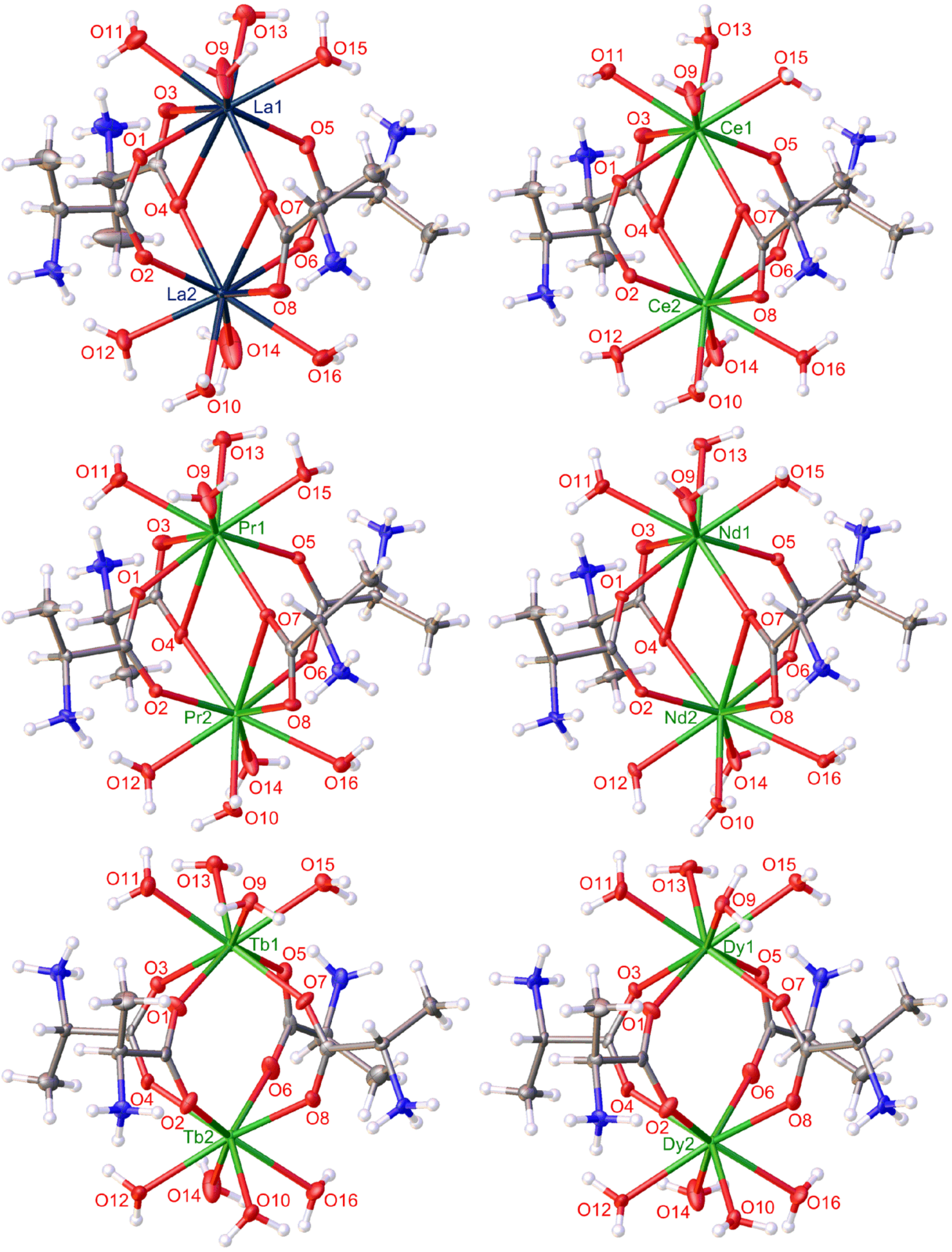

Figure S1. Molecular structures of cationic dimeric complexes $[Ln(H_2O)_4(L\text{-}Ala)_2]_2^{6+}$ obtained from low temperature (100 K) measurements. Displacement ellipsoids are drawn at the 50% probability level. Perchlorate anions are omitted for clarity.

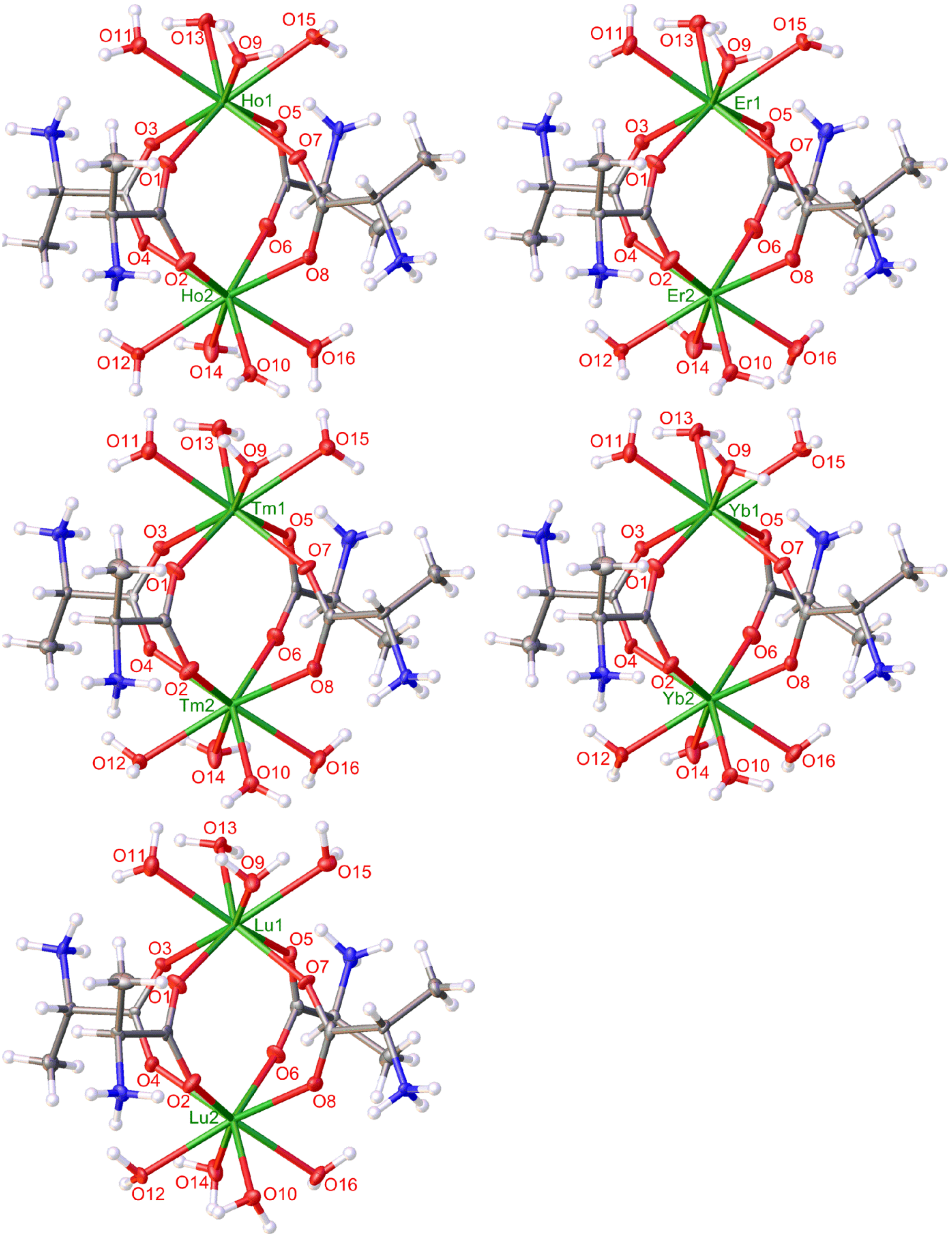

Figure S1. continued.

Table S4. Selected bond lengths and distances [Å] for dimeric lanthanide complexes with L-alanine obtained from measurements at 100 K.

Ln	La	Ce	Pr	Nd	Tb	Dy	Ho	Er	Tm	Yb	Lu
Ln1−O1	2.478(7)	2.461(4)	2.446(5)	2.427(5)	2.313(6)	2.303(5)	2.295(5)	2.284(4)	2.279(6)	2.266(5)	2.254(7)
Ln1−O3	2.504(8)	2.474(4)	2.450(5)	2.430(5)	2.337(5)	2.318(5)	2.310(5)	2.298(4)	2.291(7)	2.275(5)	2.276(7)
Ln1−O5	2.499(7)	2.485(4)	2.467(4)	2.445(5)	2.357(6)	2.341(5)	2.328(6)	2.324(4)	2.315(6)	2.306(5)	2.294(7)
Ln1−O7	2.450(7)	2.425(4)	2.412(4)	2.386(5)	2.299(6)	2.286(5)	2.276(5)	2.266(4)	2.259(6)	2.251(5)	2.240(7)
Ln1···O2	3.987(6)	3.976(3)	3.959(4)	3.938(4)	4.080(6)	4.029(6)	4.013(6)	4.002(5)	4.004(7)	3.986(5)	3.969(7)
Ln1−O4	2.875(7)	2.884(4)	2.885(5)	2.865(5)	3.774(8)	3.767(7)	3.766(8)	3.755(5)	3.750(9)	3.745(7)	3.741(9)
Ln1···O6	3.911(8)	3.909(5)	3.899(7)	3.875(7)	3.383(6)	3.430(5)	3.437(6)	3.447(4)	3.431(7)	3.452(5)	3.451(7)
Ln1···O8	4.622(8)	4.585(4)	4.577(5)	4.546(5)	4.049(5)	4.051(4)	4.057(5)	4.047(3)	4.026(5)	4.026(4)	4.036(5)
Ln2−O2	2.470(7)	2.439(4)	2.423(4)	2.405(5)	2.327(6)	2.304(5)	2.303(6)	2.292(4)	2.279(7)	2.257(5)	2.260(7)
Ln2−O4	2.486(7)	2.456(4)	2.425(4)	2.399(5)	2.365(5)	2.342(5)	2.333(5)	2.327(4)	2.313(6)	2.297(5)	2.297(7)
Ln2−O6	2.472(7)	2.438(4)	2.419(5)	2.399(5)	2.285(6)	2.267(5)	2.255(5)	2.242(4)	2.236(6)	2.232(5)	2.223(7)
Ln2−O8	2.548(8)	2.528(4)	2.519(4)	2.497(5)	2.381(5)	2.365(5)	2.346(5)	2.341(4)	2.330(7)	2.320(5)	2.305(7)
Ln2···O1	3.886(8)	3.836(5)	3.820(7)	3.793(7)	3.608(6)	3.631(5)	3.634(5)	3.629(4)	3.623(6)	3.627(5)	3.625(7)
Ln2···O3	4.604(8)	4.561(5)	4.528(5)	4.500(6)	3.966(5)	3.960(4)	3.940(4)	3.938(3)	3.928(5)	3.918(4)	3.906(5)
Ln2···O5	3.993(6)	3.958(3)	3.937(4)	3.911(4)	4.263(7)	4.226(6)	4.207(6)	4.197(4)	4.190(8)	4.175(6)	4.153(8)
Ln2−O7	2.772(7)	2.763(4)	2.746(4)	2.736(5)	3.734(8)	3.710(7)	3.692(8)	3.693(6)	3.693(9)	3.683(7)	3.665(9)
Ln1−O9	2.535(7)	2.494(4)	2.468(5)	2.450(6)	2.383(5)	2.394(5)	2.361(6)	2.377(5)	2.345(7)	2.322(5)	2.321(7)
Ln1−O11	2.563(7)	2.526(4)	2.509(4)	2.468(5)	2.420(6)	2.401(5)	2.376(6)	2.381(4)	2.375(7)	2.352(5)	2.345(7)
Ln1−O13	2.577(7)	2.558(4)	2.538(4)	2.513(5)	2.423(6)	2.513(5)	2.395(5)	2.490(4)	2.373(6)	2.351(5)	2.335(7)
Ln1−O15	2.563(8)	2.528(4)	2.508(4)	2.471(5)	2.535(5)	2.373(5)	2.501(6)	2.347(4)	2.484(6)	2.474(5)	2.453(7)
Ln2−O10	2.570(7)	2.537(4)	2.522(4)	2.494(5)	2.394(6)	2.421(5)	2.368(6)	2.403(4)	2.344(6)	2.343(5)	2.338(7)
Ln2−O12	2.559(8)	2.541(4)	2.525(4)	2.502(5)	2.440(5)	2.336(5)	2.409(6)	2.319(4)	2.398(6)	2.389(5)	2.391(7)
Ln2−O14	2.463(8)	2.453(5)	2.449(5)	2.433(5)	2.358(6)	2.402(5)	2.332(6)	2.382(4)	2.321(7)	2.295(6)	2.289(7)
Ln2−O16	2.657(7)	2.654(4)	2.639(4)	2.617(5)	2.418(5)	2.378(5)	2.391(6)	2.353(4)	2.374(6)	2.365(5)	2.365(7)
Ln1···Ln2	4.213(1)	4.171(1)	4.153(1)	4.122(1)	4.360(1)	4.364(1)	4.354(1)	4.348(1)	4.328(1)	4.332(1)	4.326(1)

Table S5. Selected bond lengths and distances [Å] for dimeric lanthanide complexes with D-alanine obtained from measurements at 100 K.

Ln	La	Ce	Pr	Nd	Tb	Dy	Ho	Er	Tm	Yb	Lu
Ln1−O1	2.470(7)	2.439(4)	2.428(8)	2.404(6)	2.303(5)	2.284(6)	2.284(4)	2.268(5)	2.255(6)	2.249(5)	2.233(5)
Ln1−O3	2.549(8)	2.535(5)	2.518(8)	2.500(6)	2.364(5)	2.352(6)	2.338(4)	2.326(5)	2.315(6)	2.300(5)	2.299(5)
Ln1−O5	2.457(8)	2.438(4)	2.425(8)	2.399(6)	2.332(5)	2.312(6)	2.311(4)	2.305(5)	2.292(6)	2.281(5)	2.270(5)
Ln1−O7	2.480(7)	2.453(5)	2.441(9)	2.402(6)	2.322(5)	2.309(6)	2.304(4)	2.288(5)	2.279(6)	2.272(5)	2.261(5)
Ln1···O2	4.001(7)	3.956(4)	3.947(8)	3.922(6)	4.058(5)	4.066(5)	4.063(4)	4.053(5)	4.029(6)	4.036(5)	4.024(5)
Ln1−O4	2.767(7)	2.758(4)	2.751(9)	2.746(6)	3.405(6)	3.436(6)	3.451(5)	3.458(6)	3.428(7)	3.460(6)	3.461(5)
Ln1···O6	3.873(7)	3.837(4)	3.821(8)	3.808(6)	3.776(4)	3.774(6)	3.766(4)	3.760(5)	3.746(6)	3.751(5)	3.749(5)
Ln1···O8	4.597(8)	4.558(5)	4.505(10)	4.497(7)	4.075(5)	4.033(6)	4.011(4)	3.996(5)	4.001(6)	3.989(6)	3.972(5)
Ln2−O2	2.509(7)	2.485(4)	2.464(8)	2.461(6)	2.381(5)	2.374(6)	2.355(4)	2.343(5)	2.339(6)	2.324(5)	2.320(5)
Ln2−O4	2.457(7)	2.425(5)	2.400(8)	2.394(6)	2.275(5)	2.261(6)	2.256(4)	2.248(5)	2.233(6)	2.230(5)	2.214(5)
Ln2−O6	2.485(7)	2.458(5)	2.435(9)	2.427(6)	2.358(5)	2.348(6)	2.336(4)	2.324(5)	2.317(6)	2.308(5)	2.304(5)
Ln2−O8	2.499(7)	2.469(5)	2.439(10)	2.439(6)	2.325(5)	2.303(6)	2.304(4)	2.294(5)	2.276(6)	2.265(5)	2.256(5)
Ln2···O1	3.926(7)	3.906(4)	3.897(7)	3.877(6)	3.731(5)	3.723(6)	3.702(5)	3.702(5)	3.691(6)	3.680(6)	3.675(5)
Ln2···O3	4.620(7)	4.586(5	4.563(8)	4.549(6)	4.265(5)	4.237(6)	4.222(4)	4.205(5)	4.191(6)	4.172(5)	4.156(4)
Ln2···O5	3.979(8)	3.979(4)	3.945(9)	3.939(6)	3.974(4)	3.949(5)	3.950(4)	3.949(5)	3.930(6)	3.916(5)	3.912(4)
Ln2−O7	2.878(7)	2.894(5)	2.867(9)	2.878(6)	3.608(5)	3.645(6)	3.640(5)	3.644(5)	3.611(6)	3.632(6)	3.618(5)
Ln1−O9	2.457(8)	2.454(5)	2.455(8)	2.441(6)	2.388(5)	2.368(6)	2.365(5)	2.359(5)	2.347(6)	2.327(6)	2.324(5)
Ln1−O11	2.660(7)	2.650(5)	2.639(8	2.612(6)	2.533(5)	2.514(6)	2.502(4)	2.490(5)	2.477(6)	2.471(6)	2.464(5)
Ln1−O13	2.566(7)	2.541(5)	2.501(9)	2.506(6)	2.421(5)	2.403(6)	2.396(4)	2.386(5)	2.373(6)	2.351(5)	2.344(5)
Ln1−O15	2.556(7)	2.550(5)	2.523(9)	2.506(6)	2.414(5)	2.388(6)	2.388(5)	2.377(5)	2.369(6)	2.349(6)	2.349(5)
Ln2−O10	2.574(7)	2.550(4)	2.549(8)	2.515(6)	2.400(5)	2.387(6)	2.375(4)	2.357(5)	2.347(6)	2.341(5)	2.317(5)
Ln2−O12	2.558(8)	2.523(5)	2.503(9)	2.479(6)	2.420(5)	2.416(6)	2.397(5)	2.390(5)	2.374(6)	2.374(5)	2.361(5)
Ln2−O14	2.523(7)	2.496(5)	2.477(9)	2.459(6)	2.360(5)	2.342(6)	2.334(5)	2.322(6)	2.306(7)	2.298(6)	2.284(5)
Ln2−O16	2.569(7)	2.531(5)	2.504(8)	2.481(6)	2.445(5)	2.434(6)	2.419(4)	2.412(5)	2.398(6)	2.397(5)	2.381(5)
Ln1···Ln2	4.210(1)	4.171(1)	4.151(1)	4.134(1)	4.364(2)	4.366(1)	4.364(1)	4.357(1)	4.323(1)	4.336(1)	4.328(1)

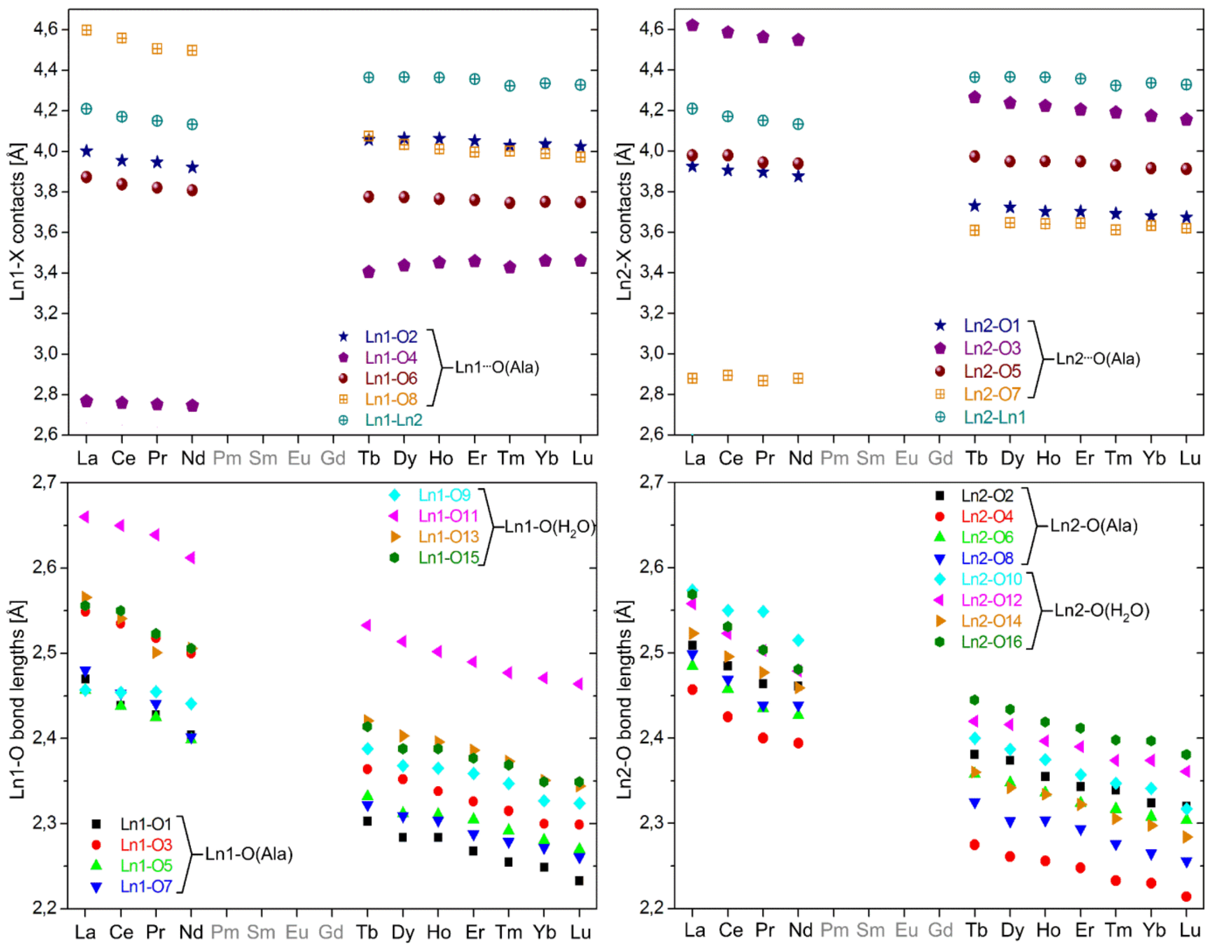

Figure S2. Graphical distribution of bond lengths and distances obtained from measurements at 100 K for both lanthanide atoms in dimeric complexes $[Ln(H_2O)_4(D\text{-}Ala)_2]_2(ClO_4)_6$.

Table S6. Crystal data and structure refinement details for studied L-alanine complexes with light lanthanides obtained from measurements at 292 K.

Ln	La	Ce	Pr	Nd	Sm	Eu	Gd
Chemical formula	$C_{12}H_{44}Cl_6N_4O_{40}La_2$	$C_{12}H_{44}Cl_6N_4O_{40}Ce_2$	$C_{12}H_{44}Cl_6N_4O_{40}Pr_2$	$C_{12}H_{44}Cl_6N_4O_{40}Nd_2$	$C_{12}H_{44}Cl_6N_4O_{40}Sm_2$	$C_{12}H_{44}Cl_6N_4O_{40}Eu_2$	$C_{12}H_{44}Cl_6N_4O_{40}Gd_2$
Formula weight	1375.03	1377.45	1379.03	1385.69	1397.91	1401.13	1411.71
λ (Mo Kα) (Å)	0.71073	0.71073	0.71073	0.71073	0.71073	0.71073	0.71073
Crystal system	triclinic	triclinic	triclinic	triclinic	triclinic	triclinic	triclinic
Space group	*P* 1	*P* 1	*P* 1	*P* 1	*P* 1	*P* 1	*P* 1
a (Å)	11.00132(17)	11.0181(5)	11.0297(4)	11.0332(3)	11.0709(2)	11.06390(18)	11.0619(3)
b (Å)	11.2334(2)	11.2195(4)	11.1779(4)	11.1617(3)	11.0999(3)	11.0785(2)	11.0709(2)
c (Å)	11.4433(2)	11.4347(4)	11.3992(3)	11.3917(2)	11.3723(2)	11.3554(2)	11.3472(2)
α (°)	78.6854(17)	78.675(3)	78.620(3)	78.5717(17)	78.4238(19)	78.3972(16)	64.6928(19)
β (°)	65.4734(17)	65.277(4)	65.162(3)	65.081(2)	64.803(2)	64.7353(17)	78.3737(19)
γ (°)	65.5243(18)	65.578(4)	65.574(3)	65.627(2)	65.680(2)	65.7303(17)	65.743(2)
V (Å^3)	1170.48(4)	1168.62(9)	1160.77(8)	1158.39(5)	1151.77(5)	1146.91(4)	1144.79(5)
Z	1	1	1	1	1	1	1
$D_{calc.}$ (g·cm^{-3})	1.951	1.957	1.973	1.986	2.015	2.029	2.048
μ (mm^{-1})	2.257	2.380	2.534	2.677	2.988	3.175	3.338
F(000)	680	682	684	686	690	692	694
Crystal size (mm)	0.15 x 0.12 x 0.04	0.18 x 0.16 x 0.10	0.16 x 0.10 x 0.08	0.18 x 0.12 x 0.06	0.30 x 0.25x 0.15	0.15 x 0.15 x 0.05	0.22 x 0.16 x 0.12
Reflections collected	66244	56550	25221	44223	78442	63077	62577
Unique reflections	13042	11850	11174	12338	12866	12807	12178
Reflections $I > 2\sigma(I)$	12075	11019	10124	11461	11979	11870	11217
R_{int}	0.0338	0.0384	0.0301	0.0290	0.0358	0.0339	0.0368
Restraints/parameters	4 / 594	3 / 635	3 / 588	3 / 664	4 / 663	4 / 664	3 / 632
Goodness-of-fit	1.104	1.076	1.062	1.082	1.069	1.100	1.065
R_1, wR_2 ($I > 2\sigma(I)$)[a]	0.0332, 0.0850	0.0276, 0.0689	0.0347, 0.0852	0.0269, 0.0660	0.0271, 0.0672	0.0481, 0.1233	0.0282, 0.0693
R_1, wR_2 (all data)[a]	0.0372, 0.0887	0.0312, 0.0721	0.0402, 0.0899	0.0306, 0.0693	0.0305, 0.0703	0.0513, 0.1269	0.0324, 0.0730
Peak/hole (e·Å^{-3})	0.683/−0.639	0.504/−0.739	0.865/−0.600	0.647/−0.717	0.793/−0.600	0.937/−1.090	0.932/−0.603

Table S7. Comparison of bond lengths and distances [Å] for dimeric L-alanine complexes with light lanthanides obtained from crystal structure measurements carried out at 100 and 292 K. The highest differences in distances between both measurements for the respective lanthanide are marked in red.

**Ln**	**La**	**La**	**Ce**	**Ce**	**Pr**	**Pr**	**Nd**	**Nd**
**T [K]**	**100**	**292**	**100**	**292**	**100**	**292**	**100**	**292**
Ln1–O1	2.478(7)	2.460(7)	2.461(4)	2.453(7)	2.446(5)	2.466(11)	2.427(5)	2.397(7)
Ln1–O3	2.504(8)	2.431(10)	2.474(4)	2.439(9)	2.450(5)	2.407(13)	2.430(5)	2.413(8)
Ln1–O5	2.499(7)	2.478(9)	2.485(4)	2.489(8)	2.467(4)	2.459(11)	2.445(5)	2.442(9)
Ln1–O7	2.450(7)	2.479(10)	2.425(4)	2.421(9)	2.412(4)	2.369(12)	2.386(5)	2.349(9)
Ln1···O2	3.987(6)	4.048(9)	3.976(3)	4.040(8)	3.959(4)	4.049(12)	3.938(4)	4.020(8)
Ln1–O4	2.875(7)	3.278(17)	2.884(4)	3.403(13)	2.885(5)	3.416(17)	2.865(5)	3.492(13)
Ln1···O6	3.911(8)	3.828(13)	3.909(5)	3.871(9)	3.899(7)	3.842(12)	3.875(7)	3.851(10)
Ln1···O8	4.622(8)	4.579(11)	4.585(4)	4.449(11)	4.577(5)	4.374(15)	4.546(5)	4.337(11)
Ln2–O2	2.470(7)	2.505(9)	2.439(4)	2.436(8)	2.423(4)	2.421(11)	2.405(5)	2.412(7)
Ln2–O4	2.486(7)	2.399(10)	2.456(4)	2.397(8)	2.425(4)	2.381(13)	2.399(5)	2.364(9)
Ln2–O6	2.472(7)	2.445(11)	2.438(4)	2.406(8)	2.419(5)	2.361(10)	2.399(5)	2.394(8)
Ln2–O8	2.548(8)	2.531(11)	2.528(4)	2.468(8)	2.519(4)	2.444(13)	2.497(5)	2.410(8)
Ln2···O1	3.886(8)	3.936(9)	3.836(5)	3.875(8)	3.820(7)	3.860(11)	3.793(7)	3.842(8)
Ln2···O3	4.604(8)	4.431(10)	4.561(5)	4.408(11)	4.528(5)	4.336(15)	4.500(6)	4.295(10)
Ln2···O5	3.993(6)	4.057(8)	3.958(3)	4.055(8)	3.937(4)	4.031(11)	3.911(4)	4.061(9)
Ln2–O7	2.772(7)	3.016(11)	2.763(4)	3.108(12)	2.746(4)	3.320(19)	2.736(5)	3.313(13)
Ln1–O9	2.535(7)	2.471(13)	2.494(4)	2.487(9)	2.468(5)	2.453(13)	2.450(6)	2.458(9)
Ln1–O11	2.563(7)	2.623(10)	2.526(4)	2.566(8)	2.509(4)	2.611(12)	2.468(5)	2.562(9)
Ln1–O13	2.577(7)	2.548(10)	2.558(4)	2.514(8)	2.538(4)	2.511(11)	2.513(5)	2.475(9)
Ln1–O15	2.563(8)	2.581(10)	2.528(4)	2.533(9)	2.508(4)	2.537(11)	2.471(5)	2.501(7)
Ln2–O10	2.570(7)	2.573(10)	2.537(4)	2.548(9)	2.522(4)	2.481(12)	2.494(5)	2.501(8)
Ln2–O12	2.559(8)	2.542(9)	2.541(4)	2.555(9)	2.525(4)	2.509(12)	2.502(5)	2.514(8)
Ln2–O14	2.463(8)	2.531(10)	2.453(5)	2.484(8)	2.449(5)	2.484(13)	2.433(5)	2.445(9)
Ln2–O16	2.657(7)	2.631(11)	2.654(4)	2.647(8)	2.639(4)	2.565(10)	2.617(5)	2.579(7)
Ln1···Ln2	4.213(1)	4.375(1)	4.171(1)	4.386(1)	4.153(1)	4.404(1)	4.122(1)	4.406(1)

Table S8. Selected bond lengths and distances [Å] for dimeric lanthanide complexes with L-alanine obtained from measurements at 292 K.

Ln	La	Ce	Pr	Nd	Sm	Eu	Gd
Ln1−O1	2.460(7)	2.453(7)	2.466(11)	2.397(7)	2.366(8)	2.369(12)	2.367(10)
Ln1−O3	2.431(10)	2.439(9)	2.407(13)	2.413(8)	2.369(8)	2.351(14)	2.346(9)
Ln1−O5	2.478(9)	2.489(8)	2.459(11)	2.442(9)	2.387(9)	2.381(15)	2.365(11)
Ln1−O7	2.479(10)	2.421(9)	2.369(12)	2.349(9)	2.303(9)	2.295(13)	2.271(11)
Ln1···O2	4.048(9)	4.040(8)	4.049(12)	4.020(8)	4.007(8)	4.005(13)	3.986(9)
Ln1−O4	3.278(17)	3.403(13)	3.416(17)	3.492(13)	3.563(11)	3.578(19)	3.606(14)
Ln1···O6	3.828(13)	3.871(9)	3.842(12)	3.851(10)	3.832(10)	3.793(17)	3.802(12)
Ln1···O8	4.579(11)	4.449(11)	4.374(15)	4.337(11)	4.253(11)	4.224(17)	4.185(12)
Ln2−O2	2.505(9)	2.436(8)	2.421(11)	2.412(7)	2.392(9)	2.373(13)	2.364(10)
Ln2−O4	2.399(10)	2.397(8)	2.381(13)	2.364(9)	2.349(9)	2.337(14)	2.331(9)
Ln2−O6	2.445(11)	2.406(8)	2.361(10)	2.394(8)	2.362(9)	2.323(14)	2.312(10)
Ln2−O8	2.531(11)	2.468(8)	2.444(13)	2.410(8)	2.389(9)	2.379(14)	2.367(10)
Ln2···O1	3.936(9)	3.875(8)	3.860(11)	3.842(8)	3.816(8)	3.828(14)	3.805(10)
Ln2···O3	4.431(10)	4.408(11)	4.336(15)	4.295(10)	4.198(10)	4.176(15	4.164(10)
Ln2···O5	4.057(8)	4.055(8)	4.031(11)	4.061(9)	4.056(10)	4.038(15)	4.028(11)
Ln2−O7	3.016(11)	3.108(12)	3.320(19)	3.313(13)	3.479(14)	3.521(15)	3.523(15)
Ln1−O9	2.471(13)	2.487(9)	2.453(13)	2.458(9)	2.427(9)	2.412(15)	2.443(12)
Ln1−O11	2.623(10)	2.566(8)	2.611(12)	2.562(9)	2.521(9)	2.489(14	2.497(9)
Ln1−O13	2.548(10)	2.514(8)	2.511(11)	2.475(9)	2.453(8)	2.439(13)	2.402(10)
Ln1−O15	2.581(10)	2.533(9)	2.537(11)	2.501(7)	2.449(8)	2.449(12)	2.436(10)
Ln2−O10	2.573(10)	2.548(9)	2.481(12)	2.501(8)	2.451(9)	2.418(14)	2.424(10)
Ln2−O12	2.542(9)	2.555(9)	2.509(12)	2.514(8)	2.516(10)	2.479(15)	2.471(10)
Ln2−O14	2.531(10)	2.484(8)	2.484(13)	2.445(9)	2.417(10)	2.413(15)	2.360(10)
Ln2−O16	2.631(11)	2.647(8)	2.565(10)	2.579(7)	2.567(9)	2.572(14)	2.541(11)
Ln1···Ln2	4.375(1)	4.386(1)	4.404(1)	4.406(1)	4.421(1)	4.417(1)	4.416(1)

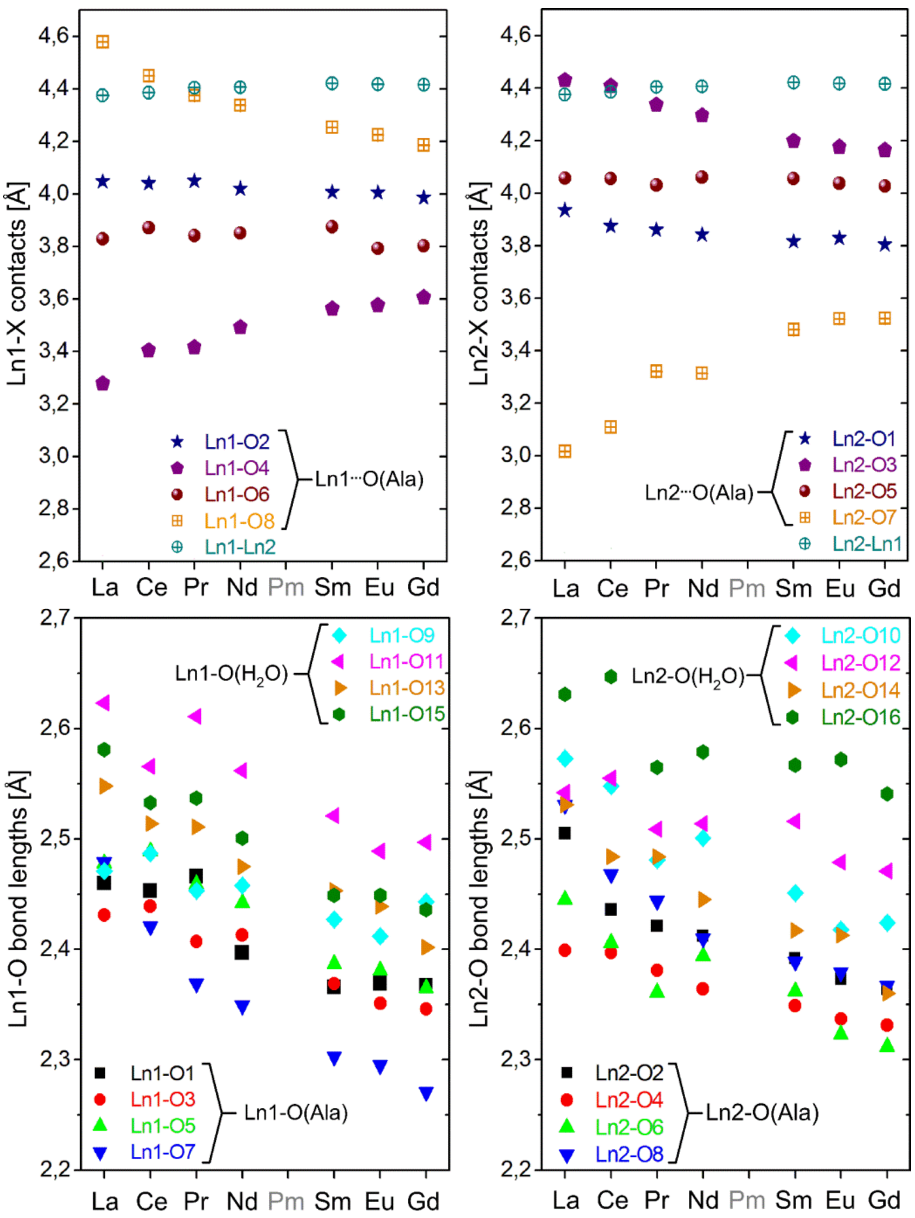

Figure S3. Graphical distribution of bond lengths and distances obtained from measurements at 292 K for both lanthanide atoms in dimeric complexes $[Ln(H_2O)_4(L\text{-}Ala)_2]_2(ClO_4)_6$.

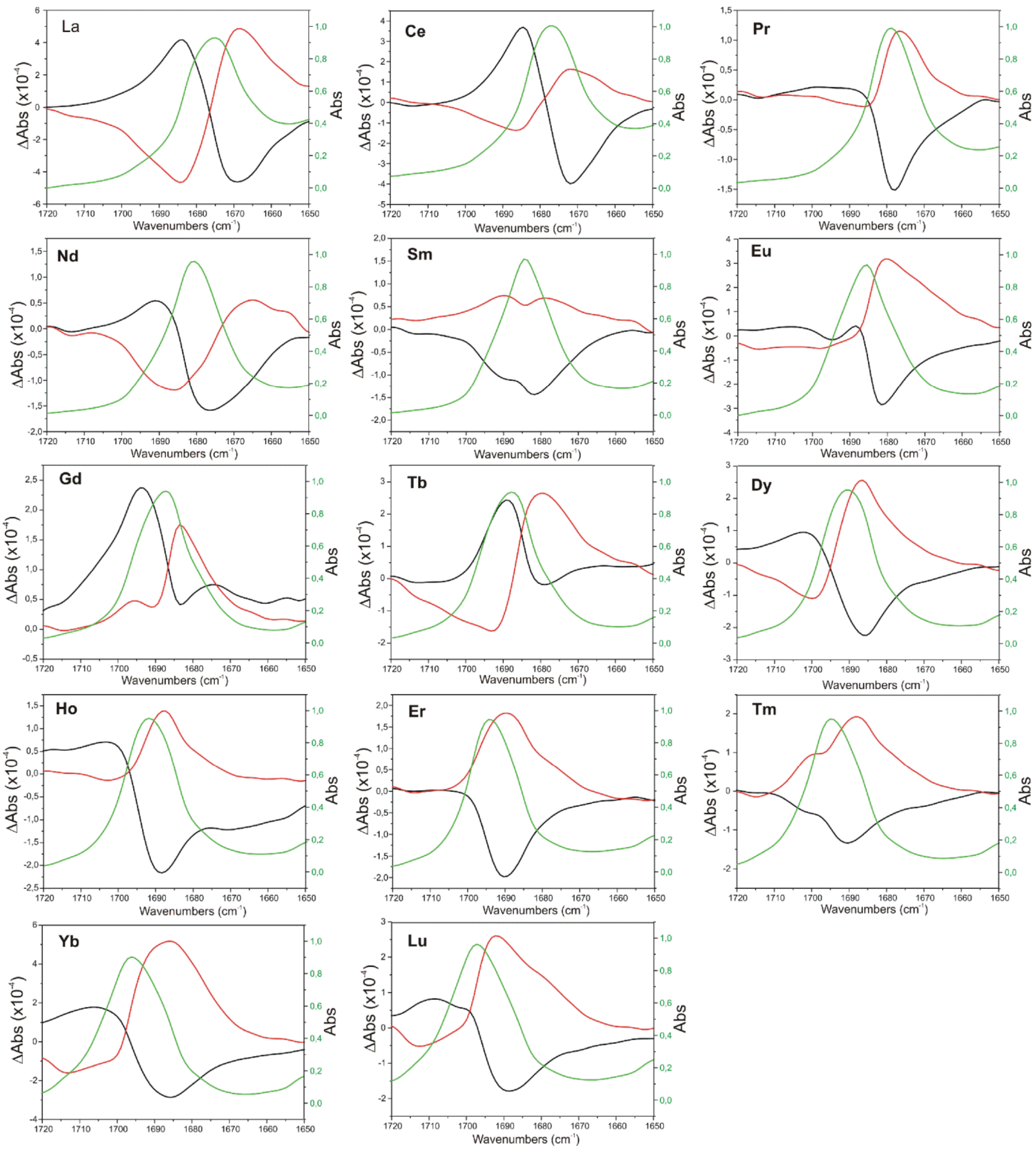

Figure S4. Juxtaposition of the experimental solid-state VCD spectra of L-Ala (black) and D-Ala (red) dimeric lanthanide complexes with their IR counterparts (green) in ν(C=O) vibrational range (measured in KBr pellets).

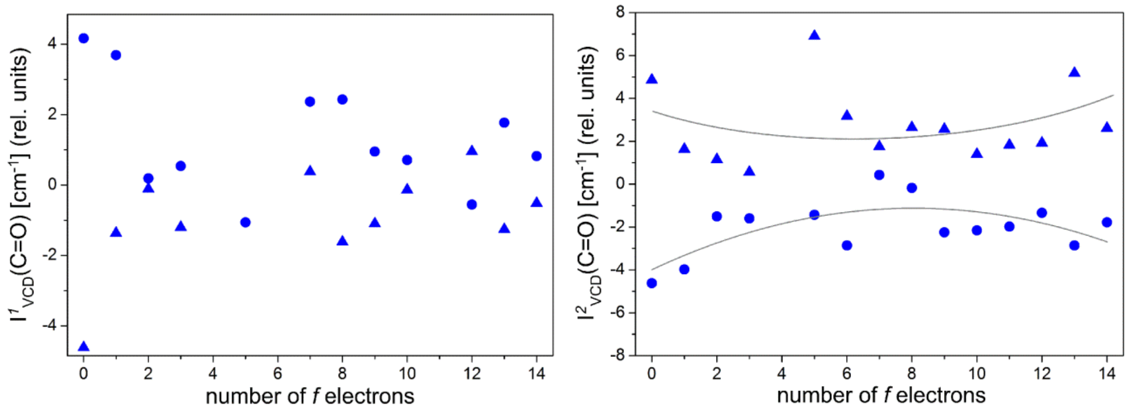

Figure S5. Variation of the VCD intensity of the ν^1(C=O) and ν^2(C=O) VCD bands with the number of 4*f* electrons in the Ln complexes with L- (circles) and D-alanine (triangles).

Table S9. Comparison of distances (Å) in $[Lu(H_2O)_4(L\text{-}Ala)_2]_2(ClO_4)_6$ and $[Lu(H_2O)_4(L\text{-}Ala)_2]_2^{6+}$ systems obtained with X-ray measurements and calculations at different computational levels, respectively. The best agreement with the experimental data is indicated in grey.

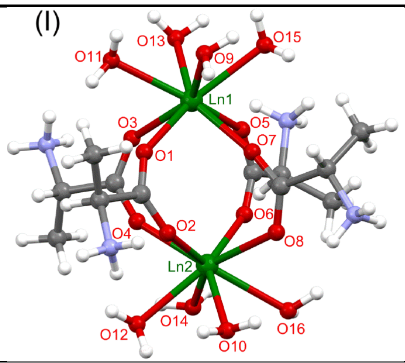

X-ray		Calculations (B3LYP/SDD(Ln)+PP(Ln))*				
			6-31G**	6-31G** PCM	TZVP	TZVP PCM
Lu1-Lu2	4.326	Lu1-Lu2	4.3967	4.267	4.416	4.273
Lu1-O7	2.240	Lu1-O40	2.2808	2.263	2.291	2.268
Lu1-O5	2.295	Lu1-O24	2.3740	2.287	2.382	2.313
Lu1-O1	2.253	Lu1-O15	2.2893	2.263	2.303	2.273
Lu1-O3	2.276	Lu1-O41	2.3639	2.298	2.380	2.307
Lu2-O8	2.305	Lu2-O42	2.3739	2.288	2.382	2.302
Lu2-O6	2.224	Lu2-O23	2.2811	2.260	2.290	2.270
Lu2-O2	2.260	Lu2-O16	2.3639	2.290	2.380	2.303
Lu2-O4	2.297	Lu2-O43	2.2892	2.269	2.303	2.276
Lu-H2O		Lu-H2O				
Lu1-O15	2.452	Lu1-O12	2.4495	2.436	2.452	2.419
Lu1-O9	2.321	Lu1-O29	2.4134	2.373	2.426	2.397
Lu1-O13	2.335	Lu1-O3	2.3905	2.351	2.415	2.387
Lu1-O11	2.345	Lu1-O20	2.4254	2.432	2.439	2.390
Lu2-O16	2.365	Lu2-O17	2.4492	2.392	2.452	2.413
Lu2-O10	2.338	Lu2-O6	2.3904	2.384	2.414	2.395
Lu2-O14	2.289	Lu2-O76	2.4133	2.398	2.426	2.405
Lu2-O12	2.391	Lu2-O9	2.4255	2.391	2.440	2.389

* the atom labels in calculations are concordant with the XYZ data gathered at the end of SM in Table S13

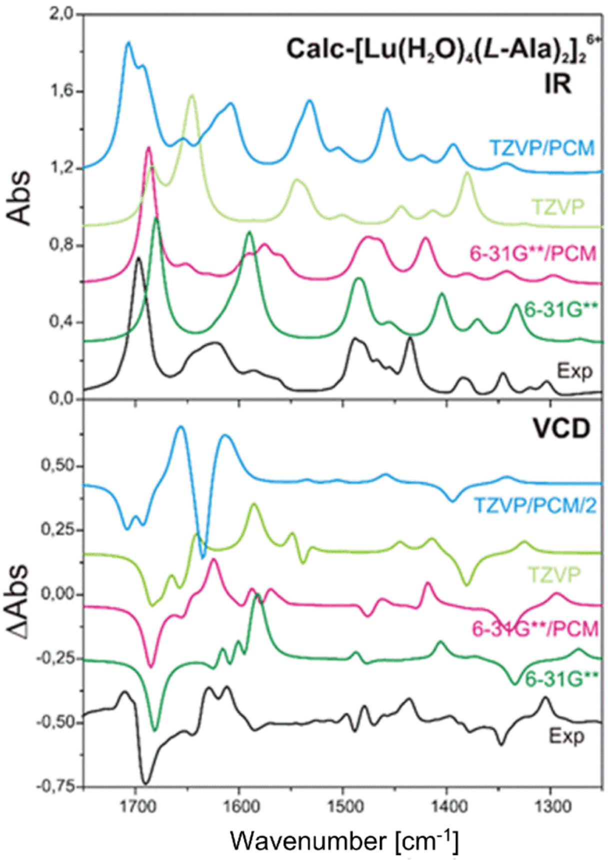

Figure S6. Comparison of the experimental solid-state IR and VCD spectra of $[Lu(H_2O)_4(L\text{-}Ala)_2]_2(ClO_4)_6$ with the calculated ones obtained for singlet state of $[Lu(H_2O)_4(L\text{-}Ala)_2]_2^{6+}$ with the B3LYP functional, different basis sets for C,O,N,H atoms, SDD(Lu) basis sets and pseudopotential for Lu, and presence or absence of the PCM(water) solvation model. The calculated spectra are shifted by 50 cm^{-1} towards lower wavenumbers.

Table S10. The total (E, au) and relative (ΔE, kcal/mol) energies referred to the most stable form for different multiplicity states of the $[Ln(H_2O)_4(L\text{-}Ala)_2]_2^{6+}$ systems obtained at the B3LYP/6-31G**(C,N,O,H)/SDD(Ln)+PP(Ln)/PCM(H_2O) level. M stands for multiplicity (M1–singlet, M3–triplet, M5 – quintet *etc.*), nc–not converged.

**$(Ln^{3+})_2$**	**E**	**ΔE**
Pr_M1*	-2940.333519	21.30
Pr_M3*	-2940.350237	10.81
Pr_M5*	-2940.367464	0.00
Nd_M1	-3028.895414	180.25
Nd_M3	-3029.151032	19.85
Nd_M5*	-3029.143124	24.81
Nd_M7	-3029.182657	0.00
Tb_M1-M13	nc	nc
Dy_M1	-3675.442824	801.20
Dy_M3	-3676.602296	73.62
Dy_M5,M7	nc	
Dy_M9	-3676.719612	0.00
Ho_M1*	-3802.740549	0.00
Ho_M1-M9	nc	
Er_M1	-3936.911740	0.00
Er_M3-M7	nc	
Tm_M1*	-4077.636991	105.91
Tm_M3	-4077.783177	14.18
Tm_M5*	-4077.805776	0.00
Yb_M1*	-4224.865456	87.34
Yb_M3*	-4225.004634	0.00
Lu_M1	-4378.913063	

* presence of low imaginary frequencies connected with water librations.

Table S11. Comparison of distances (Å) in $[Yb(H_2O)_4(L\text{-}Ala)_2]_2(ClO_4)_6$ and $[Yb(H_2O)_4(L\text{-}Ala)_2]_2^{6+}$ systems obtained respectively with X-ray measurements and calculations performed for singlet and triplet states at the B3LYP/6-31G**(C,O,N,H)/SDD(Yb)+PP(Yb)/PCM(water) level. The best agreement with the experimental data is indicated in grey.

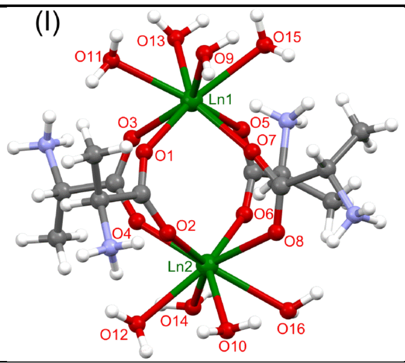

X-ray		Calculations*		
			Singlet	Triplet
Yb1-Yb2	4.332	Yb1-Yb2	4.378	4.224
Yb1-O7	2.251	Yb1-O40	2.292	2.275
Yb1-O5	2.306	Yb1-O24	2.343	2.306
Yb1-O1	2.266	Yb1-O15	2.316	2.289
Yb1-O3	2.275	Yb1-O41	2.336	2.300
Yb2-O8	2.319	Yb2-O42	2.334	2.305
Yb2-O6	2.232	Yb2-O23	2.287	2.272
Yb2-O2	2.257	Yb2-O16	2.328	2.297
Yb2-O4	2.297	Yb2-O43	2.297	2.272
Yb-H2O		Yb-H2O		
Yb1-O15	2.4783	Yb1-O12	2.406	2.385
Yb1-O9	2.3264	Yb1-O29	2.452	2.441
Yb1-O11	2.3486	Yb1-O3	2.463	2.435
Yb1-O13	2.3529	Yb1-O20	2.412	2.395
Yb2-O16	2.3639	Yb2-O17	2.440	2.412
Yb2-O10	2.3464	Yb2-O6	2.461	2.409
Yb2-O14	2.2938	Yb2-O76	2.433	2.412
Yb2-O12	2.3866	Yb2-O9	2.446	2.432

*the atom labels in calculations are concordant with the XYZ data gathered at the end of SM in Table S13

Table S12. Comparison of distances (Å) in $[Nd(H_2O)_4(L\text{-}Ala)_2]_2(ClO_4)_6$ and $[Nd(H_2O)_4(L\text{-}Ala)_2]_2^{6+}$ systems obtained respectively with X-ray measurements and calculations performed for different multiplicity states at the B3LYP/6-31G**(C,O,N,H)/SDD(Nd)+PP(Nd)/PCM(water) level. The best agreement with the experimental data is indicated in grey.

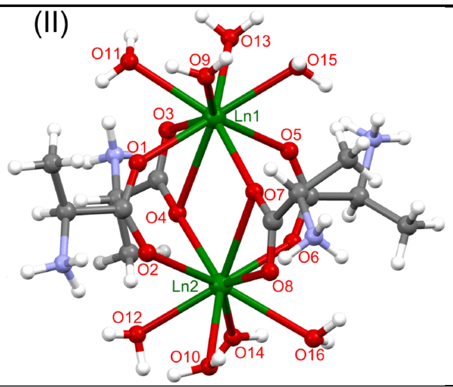

X-ray		Calculations*				
			Singlet	Triplet	Quintet	Septet
Nd1-Nd2	4.122	Nd1-Nd2	4.194	4.261	4.220	4.212
Nd1-O1	2.428	Nd1-O28	2.452	2.438	2.454	2.454
Nd2-O2	2.405	Nd2-O11	2.367	2.375	2.406	2.407
Nd1-O5	2.445	Nd1-O12	2.427	2.451	2.440	2.441
Nd2-O6	2.399	Nd2-O24	2.409	2.392	2.434	2.437
Nd1-O7	2.386	Nd1-O16	2.404	2.376	2.434	2.438
Nd2-O7	2.736	Nd2-O16	3.944	3.774	2.689	2.678
Nd1-O4	2.864	Nd1-O20	3.792	2.394	3.137	3.120
Nd2-O4	2.400	Nd2-O20	2.417	3.765	2.393	2.397
Nd1-O3	2.430	Nd1-O63	2.452	2.458	2.475	2.475
Nd2-O8	2.497	Nd2-O64	2.397	2.459	2.543	2.547
Nd-H_2O		Nd-H_2O				
Nd1-O9	2.447	Nd1-O41	2.536	2.537	2.543	2.543
Nd1-O11	2.465	Nd1-O25	2.520	2.529	2.514	2.514
Nd1-O13	2.513	Nd1-O29	2.534	2.538	2.507	2.507
Nd1-O15	2.471	Nd1-O13	2.545	2.540	2.517	2.516
Nd2-O10	2.494	Nd2-O50	2.502	2.558	2.557	2.557
Nd2-O12	2.502	Nd2-O17	2.645	2.622	2.639	2.646
Nd2-O14	2.431	Nd2-O57	2.559	2.521	2.519	2.516
Nd2-O16	2.620	Nd2-O21	2.549	2.559	2.531	2.537
		Far from the metal center				
C1-O1	1.239	C33-O28	1.256	1.256	1.261	1.261
C1-O2	1.261	C33-O11	1.265	1.264	1.262	1.261
C7-O5	1.248	C32-O12	1.262	1.260	1.265	1.265
C7-O6	1.258	C32-O24	1.257	1.258	1.256	1.256
C1-C2	1.528	C33-C35	1.534	1.534	1.534	1.534
C7-C8	1.538	C32-C44	1.537	1.537	1.538	1.538
C2-N1	1.501	C35-N7	1.510	1.508	1.509	1.509
C2-C3	1.503	C35-C59	1.527	1.527	1.527	1.527
C8-N3	1.494	C44-N3	1.511	1.512	1.512	1.512
C8-C9	1.513	C44-C46	1.526	1.531	1.530	1.530

*the atom labels in calculations are concordant with the XYZ data gathered at the end of SM in Table S13

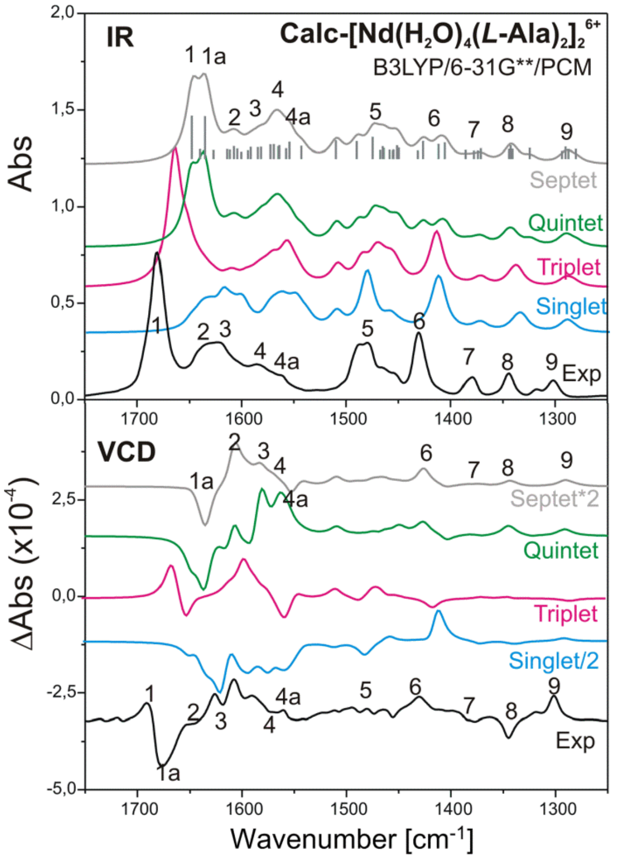

Figure S7. Comparison of the experimental solid-state IR and VCD spectra of $[Nd(H_2O)_4(L\text{-}Ala)_2]_2(ClO_4)_6$ with the calculated ones for different multiplicity states of $[Nd(H_2O)_4(L\text{-}Ala)_2]_2^{6+}$ with the B3LYP functional, 6-31G**(C,O,N,H) and SDD(Nd) basis set and pseudopotential plus PCM(water) solvation model. The calculated spectra are shifted by 50 cm^{-1} towards lower wavenumbers.

Table S13. Cartesian Coordinates of the $[Ln(H_2O)_4(L\text{-}Ala)_2]_2^{6+}$ complexes calculated at different computational levels (B3LYP/SDD(Ln)+PP(Ln)).

$\{[Lu(H_2O)_4(L\text{-}Ala)_2]_2\}^{6+}$ singlet 6-31G**(C,N,O,H)/PCM(H_2O)				$\{[Lu(H_2O)_4(L\text{-}Ala)_2]_2\}^{6+}$ singlet TZVP(C,N,O,H)/PCM(H_2O)				$\{[Lu(H_2O)_4(L\text{-}Ala)_2]_2\}^{6+}$ singlet 6-31G**(C,N,O,H)			
Lu	-2.109319	-0.352769	-0.112653	Lu	2.125549	-0.262081	0.036848	Lu	2.196447	0.090833	-0.061874
Lu	2.103892	0.314161	-0.000326	Lu	-2.117865	0.236103	0.056551	Lu	-2.196506	-0.090398	-0.061949
O	-3.995463	1.033107	-0.331773	O	3.871556	1.332864	0.360456	O	3.985485	-1.494245	-0.095179
H	-4.599246	0.993982	0.425182	H	4.431007	1.501145	-0.409815	H	4.665167	-1.469268	0.596270
H	-4.517442	0.873465	-1.132158	H	3.739716	2.174011	0.815643	H	4.208125	-2.231405	-0.682034
O	3.920202	-1.229748	0.020668	O	-3.867507	-1.392047	-0.093781	O	-3.984783	1.495626	-0.088721
H	3.769063	-2.156435	-0.213492	H	-3.734246	-2.322931	0.125342	H	-4.206763	2.234882	-0.673169
H	4.431654	-1.218203	0.843447	H	-4.431860	-1.352785	-0.877448	H	-4.664604	1.468845	0.602522
O	3.559823	0.683892	-1.860173	O	-3.519116	0.327145	1.989787	O	-3.578097	-0.357947	-2.037456
H	3.138985	0.854963	-2.715207	H	-3.121079	0.527697	2.847060	H	-3.353444	-0.866552	-2.830016
H	4.228804	-0.002402	-1.999250	H	-4.203231	-0.340694	2.127037	H	-4.472445	-0.006891	-2.171437
O	-3.503245	-0.373197	1.885259	O	3.571302	-0.112902	-1.896891	O	3.639985	0.246862	1.910859
H	-3.076267	0.021925	2.658851	H	3.244962	0.295588	-2.708965	H	3.535517	-0.179870	2.772961
H	-3.844991	-1.230208	2.177153	H	4.025420	-0.927961	-2.149570	H	4.356383	0.893835	2.008628
O	-0.726497	-1.656305	-1.341710	O	0.866288	-1.633441	1.340086	O	0.915888	1.276985	-1.543112
O	1.511566	-1.407417	-1.388812	O	-1.372726	-1.609228	1.214553	O	-1.314733	1.598614	-1.461127
O	3.460568	0.588729	1.950718	O	-3.554154	0.634000	-1.840972	O	-3.640369	-0.254578	1.909610
H	3.777315	1.502428	2.019279	H	-3.963679	1.509515	-1.863076	H	-4.357065	-0.901687	2.004229
H	3.113820	0.342403	2.819797	H	-3.277949	0.423431	-2.742194	H	-3.535741	0.167930	2.773770
O	-3.224226	-0.688468	-2.247414	O	3.438684	-0.860981	1.941987	O	3.577536	0.367041	-2.036452
H	-3.352596	-1.626594	-2.451311	H	4.120298	-0.268724	2.284775	H	4.471601	0.016121	-2.172665
H	-2.733343	-0.317934	-2.995350	H	3.011939	-1.292146	2.693874	H	3.352189	0.878655	-2.826876
O	0.751297	1.595180	1.279699	O	-0.847075	1.719911	-1.100045	O	-0.865005	-1.360804	1.285829
O	-1.489483	1.378828	1.246760	O	1.378219	1.501460	-1.259383	O	1.372919	-1.506565	1.489204
N	1.947577	-3.344783	-3.106732	N	-1.745612	-3.617201	2.881464	N	-1.286233	3.416156	-3.565809
H	2.075739	-3.780894	-4.024056	H	-1.829915	-4.173631	3.734311	H	-1.118620	4.087715	-4.331435
H	2.323693	-3.988099	-2.403074	H	-2.126189	-4.172462	2.111780	H	-1.861389	3.901281	-2.867574
H	2.513052	-2.489538	-3.062205	H	-2.337740	-2.787977	2.975507	H	-1.849373	2.651372	-3.953751
O	-3.304569	-2.402291	-0.097245	O	3.281830	-2.326802	-0.342188	O	3.443160	2.156589	-0.009319
H	-2.895082	-3.207543	0.250013	H	2.852656	-3.044247	-0.826664	H	3.389182	2.910828	0.595979
H	-4.257719	-2.564597	-0.139609	H	3.771594	-2.719264	0.392373	H	4.120683	2.375851	-0.667596
N	1.745806	-3.020050	3.359842	N	-1.663851	-2.670810	-3.710736	N	-1.283494	3.560473	3.313427
H	2.383248	-2.913085	2.563603	H	-2.335223	-2.601892	-2.940633	H	-2.009481	3.574093	2.588092
H	1.914535	-3.928148	3.801539	H	-1.824798	-3.545241	-4.213647	H	-1.164954	4.521074	3.669702
H	1.995139	-2.296300	4.040780	H	-1.866806	-1.902278	-4.353884	H	-1.642754	3.007433	4.102007
N	-1.728460	3.386039	-3.051803	N	1.670134	2.758993	3.725989	N	1.285579	-3.401335	-3.579936
H	-1.984723	3.777493	-3.962153	H	1.765444	3.085256	4.689520	H	1.117757	-4.069216	-4.348727
H	-1.834545	4.129501	-2.354442	H	1.999247	3.507587	3.111894	H	1.860476	-3.890007	-2.883955
H	-2.391471	2.640903	-2.805223	H	2.294313	1.960639	3.587562	H	1.849075	-2.634953	-3.964177
O	-0.922938	-1.486046	1.446093	O	0.916271	-1.369779	-1.529575	O	0.864672	1.355411	1.290594
O	-1.344324	1.227066	-1.596101	O	1.317199	1.198098	1.629874	O	1.314806	-1.592253	-1.468194
O	1.317830	-1.243034	1.480330	O	-1.307765	-1.099883	-1.634144	O	-1.373065	1.500163	1.496110
O	0.900596	1.404431	-1.585838	O	-0.922595	1.186474	1.744318	O	-0.915791	-1.270157	-1.547945
C	-0.246975	1.729578	-1.971344	C	0.215890	1.517988	2.144433	C	0.154129	-1.835899	-1.907090
C	-0.412173	1.929164	1.607034	C	0.284964	2.077301	-1.493313	C	0.189382	-1.853385	1.771257
C	0.424345	-1.937934	-1.750776	C	-0.274841	-2.056571	1.631507	C	-0.154235	1.844075	-1.899517
C	0.230555	-1.762452	1.855629	C	-0.214453	-1.588302	-2.017656	C	-0.189399	1.845680	1.779091
C	-0.324766	2.824509	-3.053038	C	0.243630	2.376662	3.416625	C	-0.040302	-2.932069	-2.988797
H	-0.208087	2.319602	-4.016929	H	-0.076999	1.735865	4.238993	H	-0.579515	-2.478368	-3.825642

C	0.311567	-2.881842	2.906746	C	-0.253521	-2.591137	-3.178976	C	0.038178	3.007258	2.785716
H	0.066762	-3.818523	2.397731	H	-0.040647	-3.571378	-2.751289	H	0.480923	3.831044	2.215608
C	0.496452	-3.015710	-2.844425	C	-0.310733	-3.223482	2.626400	C	0.039793	2.944692	-2.976773
H	0.126056	-2.553958	-3.764142	H	0.065562	-2.842348	3.576089	H	0.578991	2.494568	-3.815554
C	-0.300263	-4.276942	-2.527245	C	0.504618	-4.430428	2.181814	C	0.806022	4.166608	-2.453824
H	-0.259729	-4.981253	-3.361329	H	0.466062	-5.217697	2.934454	H	1.022251	4.874987	-3.259271
H	-1.341191	-3.998661	-2.360589	H	1.541581	-4.127499	2.056935	H	1.760322	3.839880	-2.038705
H	0.075088	-4.768245	-1.624925	H	0.142628	-4.827888	1.232250	H	0.247463	4.689631	-1.670688
C	-0.512407	3.165058	2.514544	C	0.332791	3.382403	-2.302329	C	-0.037529	-3.019864	2.772401
H	-0.189395	4.023605	1.919390	H	0.186229	4.192425	-1.586648	H	-0.479757	-3.841152	2.198287
C	0.322949	3.056227	3.788005	C	-0.701801	3.469866	-3.413985	C	-0.940218	-2.668202	3.958838
H	0.005696	2.204521	4.397244	H	-0.571820	2.668651	-4.142988	H	-0.495719	-1.890812	4.589113
H	0.243086	3.969764	4.381612	H	-0.637437	4.430318	-3.924705	H	-1.140998	-3.546492	4.579411
H	1.368235	2.913960	3.511200	H	-1.694408	3.381095	-2.978698	H	-1.897162	-2.303717	3.583970
N	-1.965856	3.413475	2.847864	N	1.729409	3.564633	-2.846500	N	1.284520	-3.574815	3.297374
H	-2.559699	3.131316	2.059068	H	2.411932	3.218207	-2.165900	H	2.010734	-3.583202	2.572182
H	-2.140992	4.399575	3.059362	H	1.931101	4.545629	-3.048298	H	1.166934	-4.537645	3.647886
H	-2.266702	2.866353	3.660057	H	1.871012	3.034073	-3.709511	H	1.642837	-3.026095	4.089403
C	-0.607047	-2.671856	4.107531	C	0.726163	-2.281422	-4.301458	C	0.940656	2.649228	3.970424
H	-1.635826	-2.600783	3.754784	H	1.734699	-2.265167	-3.894891	H	1.897293	2.285850	3.593700
H	-0.358027	-1.750169	4.642041	H	0.517897	-1.309958	-4.752288	H	0.495597	1.869147	4.596965
H	-0.536216	-3.514895	4.798432	H	0.679099	-3.049220	-5.073908	H	1.142125	3.524367	4.595215
C	0.703786	3.939254	-2.908927	C	-0.635119	3.617298	3.339046	C	-0.806709	-4.155969	-2.470817
H	0.595681	4.668087	-3.715459	H	-0.605705	4.167288	4.279270	H	-1.023211	-4.860956	-3.279158
H	1.703285	3.509193	-2.968347	H	-1.662112	3.311099	3.155010	H	-1.760868	-3.830756	-2.054182
H	0.606061	4.451864	-1.947398	H	-0.319711	4.277525	2.529108	H	-0.248143	-4.682364	-1.689945
O	3.117417	2.485763	0.089167	O	-3.209539	2.373865	0.208946	O	-3.442430	-2.156748	-0.018052
H	3.631615	2.701046	-0.703196	H	-3.732571	2.566870	0.997950	H	-4.120210	-2.374127	-0.676688
H	2.543950	3.242673	0.277188	H	-2.718910	3.173593	-0.021416	H	-3.387846	-2.912942	0.584747
$\{[Lu(H_2O)_4(L\text{-}Ala)_2]_2\}^{6+}$ singlet TZVP(C,N,O,H)				$\{[Yb(H_2O)_4(L\text{-}Ala)_2]_2\}^{6+}$ singlet 6-31G**(C,N,O,H)/PCM(H_2O)				$\{[Yb(H_2O)_4(L\text{-}Ala)_2]_2\}^{6+}$ triplet 6-31G**(C,N,O,H)/PCM(H_2O)			
Lu	2.196447	0.090833	-0.061874	Yb	2.193183	-0.015781	-0.241582	Yb	2.106084	0.027421	-0.275268
Lu	-2.196506	-0.090398	-0.061949	Yb	-2.157193	-0.109893	0.242752	Yb	-2.077019	-0.148760	0.285636
O	3.985485	-1.494245	-0.095179	O	3.827761	0.134657	1.594686	O	3.700419	0.032111	1.565593
H	4.665167	-1.469268	0.596270	H	4.378105	-0.654459	1.704817	H	4.214821	-0.781435	1.671464
H	4.208125	-2.231405	-0.682034	H	3.402413	0.274078	2.453147	H	3.285491	0.196104	2.425050
O	-3.984783	1.495626	-0.088721	O	3.623750	-1.954601	-0.355993	O	3.590663	-1.830692	-0.552595
H	-4.206763	2.234882	-0.673169	H	3.186008	-2.763438	-0.658352	H	3.188655	-2.594947	-0.991126
H	-4.664604	1.468845	0.602522	H	4.542417	-2.012803	-0.654078	H	4.521732	-1.814124	-0.816908
O	-3.578097	-0.357947	-2.037456	O	-3.925024	-0.233550	-1.464117	O	-3.830989	-0.377353	-1.349387
H	-3.353444	-0.866552	-2.830016	H	-3.679477	-0.198234	-2.399133	H	-3.620176	-0.348550	-2.293221
H	-4.472445	-0.006891	-2.171437	H	-4.408784	-1.064459	-1.336810	H	-4.306600	-1.207614	-1.190132
O	3.639985	0.246862	1.910859	O	-3.732819	1.747684	0.389799	O	-3.607154	1.712482	0.384618
H	3.535517	-0.179870	2.772961	H	-3.408604	2.658886	0.366676	H	-3.221445	2.599858	0.404992
H	4.356383	0.893835	2.008628	H	-4.344317	1.653191	-0.357706	H	-4.241401	1.695006	-0.347838
O	0.915888	1.276985	-1.543112	O	3.613568	1.907343	-0.511069	O	3.547083	1.924659	-0.384747
O	-1.314733	1.598614	-1.461127	H	4.098139	1.852346	-1.348537	H	4.018601	1.970292	-1.230438
O	-3.640369	-0.254578	1.909610	H	4.277701	1.948521	0.193353	H	4.222124	1.896807	0.310268
H	-4.357065	-0.901687	2.004229	O	-0.865012	-1.496505	1.540319	O	-0.855181	-1.336301	1.787846
H	-3.535741	0.167930	2.773770	O	-3.566887	-2.089517	0.519601	O	-3.456614	-2.117213	0.655168
O	3.577536	0.367041	-2.036452	H	-3.939179	-2.066326	1.414744	H	-3.863908	-2.045087	1.532735
H	4.471601	0.016121	-2.172665	H	-3.180525	-2.969227	0.405465	H	-3.032191	-2.986220	0.620376
H	3.352189	0.878655	-2.826876	O	0.816118	1.271176	-1.545576	O	0.786873	1.413326	-1.506589

O	-0.865005	-1.360804	1.285829	N	1.805442	-3.063382	3.409876	N	1.917301	-3.292315	3.056141
O	1.372919	-1.506565	1.489204	H	1.985911	-3.957690	3.875184	H	2.058508	-4.226010	3.451050
N	-1.286233	3.416156	-3.565809	H	2.040200	-2.315276	4.070107	H	2.415406	-2.623088	3.651178
H	-1.118620	4.087715	-4.331435	H	2.444081	-2.964778	2.612943	H	2.357501	-3.249557	2.130042
H	-1.861389	3.901281	-2.867574	O	1.388554	-1.363126	1.489237	O	1.328076	-1.498976	1.258678
H	-1.849373	2.651372	-3.953751	O	-1.438853	1.328695	-1.449453	O	-1.448802	1.144588	-1.515579
O	3.443160	2.156589	-0.009319	N	-1.730480	3.214854	-3.236777	N	-1.930610	3.208000	-3.070061
H	3.389182	2.910828	0.595979	H	-2.324146	2.413563	-3.468505	H	-2.540796	2.390418	-2.962446
H	4.120683	2.375851	-0.667596	H	-1.777598	3.883894	-4.011038	H	-2.133676	3.664179	-3.963882
N	-1.283494	3.560473	3.313427	H	-2.143256	3.658622	-2.410587	H	-2.178661	3.865099	-2.323651
H	-2.009481	3.574093	2.588092	N	-1.571431	-3.283488	-3.312105	N	-1.624583	-3.492052	-3.047184
H	-1.164954	4.521074	3.669702	H	-1.765102	-4.217759	-3.683032	H	-1.772510	-4.448959	-3.379423
H	-1.642754	3.007433	4.102007	H	-1.697809	-2.614240	-4.078047	H	-1.939075	-2.855252	-3.786103
N	1.285579	-3.401335	-3.579936	H	-2.269788	-3.054351	-2.595260	H	-2.222205	-3.324297	-2.230583
H	1.117757	-4.069216	-4.348727	N	1.517504	3.728199	2.744589	N	1.569651	3.718935	2.665481
H	1.860476	-3.890007	-2.883955	H	2.263506	3.057211	2.525174	H	2.272833	2.993612	2.481510
H	1.849075	-2.634953	-3.964177	H	1.725927	4.184670	3.636972	H	1.799704	4.194806	3.541985
O	0.864672	1.355411	1.290594	H	1.536285	4.448648	2.015729	H	1.639957	4.408666	1.910689
O	1.314806	-1.592253	-1.468194	O	3.118419	0.099744	-2.509773	O	3.068579	0.299857	-2.502003
O	-1.373065	1.500163	1.496110	H	3.544314	-0.652440	-2.943118	H	3.431603	-0.456283	-2.983779
O	-0.915791	-1.270157	-1.547945	H	2.473614	0.449975	-3.141028	H	2.432859	0.721674	-3.098617
C	0.154129	-1.835899	-1.907090	O	-1.302648	-1.580842	-1.346016	O	-1.198729	-1.763625	-1.091621
C	0.189382	-1.853385	1.771257	O	-3.317475	-0.084788	2.381499	O	-3.230850	-0.046738	2.400903
C	-0.154235	1.844075	-1.899517	H	-3.859600	0.715391	2.454515	H	-3.744575	0.768187	2.504040
C	-0.189399	1.845680	1.779091	H	-2.824908	-0.165347	3.210075	H	-2.695942	-0.154090	3.200218
C	-0.040302	-2.932069	-2.988797	O	1.367376	1.533901	1.310090	O	1.298091	1.444246	1.354868
H	-0.579515	-2.478368	-3.825642	C	-0.317208	1.690773	-1.883788	C	-0.375360	1.689033	-1.893836
C	0.038178	3.007258	2.785716	O	0.953455	-1.659824	-1.301417	O	0.996316	-1.520900	-1.543902
H	0.480923	3.831044	2.215608	C	0.291745	-1.819307	1.898369	C	0.298353	-1.819880	1.909173
C	0.039793	2.944692	-2.976773	O	-0.884432	1.412102	1.380136	O	-0.950671	1.543838	1.300712
H	0.578991	2.494568	-3.815554	C	0.227822	1.885633	1.710686	C	0.179234	1.927922	1.686091
C	0.806022	4.166608	-2.453824	C	-0.177758	-2.023713	-1.698219	C	-0.126718	-2.058738	-1.692302
H	1.022251	4.874987	-3.259271	C	-0.307551	2.785005	-2.973431	C	-0.485416	2.779703	-2.974523
H	1.760322	3.839880	-2.038705	H	0.046057	2.325952	-3.900220	H	-0.252531	2.303144	-3.931825
H	0.247463	4.689631	-1.670688	C	0.374798	-2.962853	2.938259	C	0.449770	-2.951592	2.939179
C	-0.037529	-3.019864	2.772401	H	0.176066	-3.890489	2.392874	H	-0.026830	-3.839045	2.512988
H	-0.479757	-3.841152	2.198287	C	-0.581896	-2.818973	4.115023	C	-0.149062	-2.633096	4.305416
C	-0.940218	-2.668202	3.958838	H	-0.472053	-3.660069	4.803688	H	-0.011214	-3.469884	4.994072
H	-0.495719	-1.890812	4.589113	H	-1.603630	-2.819549	3.734209	H	-1.219114	-2.463020	4.184022
H	-1.140998	-3.546492	4.579411	H	-0.412353	-1.887236	4.661840	H	0.299339	-1.734942	4.739803
H	-1.897162	-2.303717	3.583970	C	0.191314	3.006084	2.779623	C	0.200921	3.079724	2.709794
N	1.284520	-3.574815	3.297374	H	0.153159	2.499827	3.748830	H	0.124417	2.617143	3.698770
H	2.010734	-3.583202	2.572182	C	-0.187669	-3.193383	-2.713391	C	-0.181042	-3.241617	-2.678371
H	1.166934	-4.537645	3.647886	H	-0.055772	-4.108862	-2.128923	H	0.140470	-4.126824	-2.120929
H	1.642837	-3.026095	4.089403	C	-0.966765	3.984082	2.640163	C	-0.894049	4.122108	2.525272
C	0.940656	2.649228	3.970424	H	-0.929569	4.736600	3.431606	H	-0.814703	4.903195	3.285059
H	1.897293	2.285850	3.593700	H	-1.903898	3.435046	2.733549	H	-1.863912	3.636637	2.636679
H	0.495597	1.869147	4.596965	H	-0.951238	4.487965	1.669584	H	-0.846247	4.581670	1.533841
H	1.142125	3.524367	4.595215	C	0.554690	3.988313	-2.603467	C	0.420874	3.984061	-2.745790
C	-0.806709	-4.155969	-2.470817	H	0.567065	4.716834	-3.418126	H	0.325115	4.698805	-3.566046
H	-1.023211	-4.860956	-3.279158	H	1.575253	3.646788	-2.427805	H	1.455494	3.643178	-2.703253
H	-1.760868	-3.830756	-2.054182	H	0.190139	4.476010	-1.694917	H	0.183990	4.486943	-1.803724
H	-0.248143	-4.682364	-1.689945	C	0.871890	-3.092683	-3.802750	C	0.668733	-3.063406	-3.930504

O	-3.442430	-2.156748	-0.018052	H	0.798860	-3.941095	-4.487536	H	0.605732	-3.948793	-4.567144
H	-4.120210	-2.374127	-0.676688	H	1.857911	-3.111947	-3.339012	H	1.709061	-2.926458	-3.635428
H	-3.387846	-2.912942	0.584747	H	0.774665	-2.164271	-4.372403	H	0.353135	-2.186974	-4.504043
$\{[Nd(H_2O)_4(L\text{-}Ala)_2]_2\}^{6+}$ singlet 6-31G**(C,N,O,H)/PCM(H_2O)				$\{[Nd(H_2O)_4(L\text{-}Ala)_2]_2\}^{6+}$ triplet 6-31G**(C,N,O,H)/PCM(H_2O)				$\{[Nd(H_2O)_4(L\text{-}Ala)_2]_2\}^{6+}$ quintet 6-31G**(C,N,O,H)/PCM(H_2O)			
Nd	2.006356	-0.461076	-0.536361	Nd	1.988387	-0.338436	-0.698881	Nd	-1.828176	0.568126	-0.976469
Nd	-1.995913	0.423565	0.349894	Nd	-2.025275	0.307700	0.576140	Nd	1.792464	-0.697241	0.784009
N	2.693126	2.497304	3.260421	N	2.818340	1.943988	3.610921	N	-3.579196	-1.782046	2.947030
H	3.180938	1.662869	2.916983	H	3.017694	1.110478	4.170751	H	-3.580504	-1.040501	3.652752
H	3.093627	2.767118	4.163231	H	3.248800	2.743468	4.083586	H	-4.293452	-2.464500	3.215024
H	2.894477	3.250704	2.595288	H	3.266948	1.806667	2.694932	H	-3.839924	-1.348391	2.050692
N	-3.028370	-2.643466	-2.590641	N	-3.068568	-2.571231	-2.516143	N	3.538084	2.898416	-1.093714
H	-3.540727	-3.482762	-2.874448	H	-3.581578	-3.411992	-2.794633	H	4.045928	3.785599	-1.048031
H	-3.308780	-1.879970	-3.212795	H	-3.388080	-1.802072	-3.112070	H	4.082100	2.252154	-1.672627
H	-3.329781	-2.374952	-1.633351	H	-3.335615	-2.327125	-1.542773	H	3.501867	2.481376	-0.143613
O	-1.559458	-0.680147	-1.698413	O	-1.558536	-0.653871	-1.545065	O	2.043765	0.771866	-1.104883
O	1.673870	1.060214	1.324690	O	1.838913	0.975879	1.364165	O	-2.072604	-0.833155	1.005992
O	3.987145	1.136149	-0.522910	O	3.887688	1.346956	-0.747670	O	-3.399187	-1.283155	-1.639267
H	3.892361	2.031739	-0.169168	H	3.786034	2.218043	-0.338853	H	-3.727310	-1.911095	-0.979690
H	4.350731	1.224688	-1.417240	H	4.135708	1.502182	-1.672422	H	-4.024820	-1.307098	-2.377345
O	1.151689	1.378783	-1.827054	O	0.848870	1.580719	-1.512771	O	-0.116158	-1.159703	-1.053260
O	-3.666156	-1.613828	0.112770	O	-3.820002	-1.559173	0.165368	O	3.403136	1.300311	1.402132
H	-4.615851	-1.436474	0.199750	H	-4.672601	-1.122614	0.002631	H	4.290936	0.904059	1.417576
H	-3.449665	-2.292106	0.772594	H	-3.980303	-2.245862	0.831096	H	3.299769	1.784721	2.235462
O	-1.125576	-1.646189	1.243497	O	-0.977457	-1.774218	1.121545	O	0.323777	1.138296	1.233034
O	-2.952750	2.700010	0.983484	O	-3.016836	2.550089	1.307356	O	2.733028	-2.980941	1.339348
H	-2.405805	3.496107	0.924733	H	-2.484969	3.310749	1.030713	H	2.510446	-3.667649	0.693258
H	-3.280852	2.653723	1.895021	H	-3.292153	2.723303	2.218609	H	2.675315	-3.391429	2.213548
O	-0.459502	1.187629	2.040820	O	-0.305587	1.063629	2.057662	O	-0.156711	-1.669279	1.871021
O	2.957550	-2.686232	-1.237912	O	3.067089	-2.388049	-1.714297	O	-2.416149	2.794355	-1.986514
H	2.298667	-3.337778	-1.519587	H	2.444893	-3.074804	-1.995578	H	-1.644710	3.376207	-2.057145
H	3.534590	-3.118053	-0.591417	H	3.741048	-2.814694	-1.165401	H	-3.124053	3.306566	-1.570831
O	0.409914	-1.749233	-1.879470	O	0.401973	-1.639067	-2.016979	O	0.145262	1.819508	-1.724483
O	3.880726	-1.182456	1.008630	O	3.969298	-1.138197	0.670827	O	-4.067857	1.305934	-0.126368
H	4.570921	-0.501648	1.024054	H	4.639599	-0.454640	0.818569	H	-4.870720	0.768870	-0.180157
H	3.666271	-1.385795	1.930260	H	3.790831	-1.546484	1.529902	H	-4.080701	1.748015	0.734874
C	0.771910	1.426566	2.128346	C	0.924605	1.303040	2.167705	C	-1.405463	-1.548595	1.807843
C	-0.834540	-1.663370	-2.028095	C	-0.853195	-1.585939	-2.025303	C	1.382569	1.775964	-1.487447
C	0.167954	2.153320	-1.738500	C	-0.153500	2.338575	-1.466779	C	0.659529	-1.962336	-1.664651
C	-1.537787	-2.874744	-2.652917	C	-1.581053	-2.782088	-2.651231	C	2.153026	3.091380	-1.660774
H	-1.340212	-3.737171	-2.011362	H	-1.346317	-3.657419	-2.039733	H	1.669473	3.842648	-1.031643
C	-0.209584	-3.372278	2.590735	C	0.022657	-3.570533	2.293513	C	-0.245075	2.928716	2.710212
H	-0.670850	-4.202055	2.048482	H	-0.596542	-4.322623	1.798721	H	0.678086	3.475011	2.501600
C	0.150673	3.328335	-2.729681	C	-0.121850	3.529016	-2.436862	C	0.184065	-2.593328	-2.971840
H	-0.057064	2.913331	-3.720110	H	-0.180539	3.119088	-3.448778	H	-0.211505	-1.810365	-3.620738
O	3.186878	-0.327742	-2.777349	O	2.868353	0.107456	-3.035997	O	-1.477689	0.220177	-3.471103
H	3.454071	-1.185399	-3.139676	H	3.157298	-0.691046	-3.502354	H	-0.830868	0.853833	-3.817360
H	2.713328	0.144549	-3.477456	H	2.318249	0.613245	-3.651121	H	-2.189280	0.179647	-4.126418
C	1.209667	2.228072	3.364037	C	1.333606	2.132993	3.394539	C	-2.210614	-2.407667	2.797339
H	1.083967	1.579079	4.235012	H	0.825207	1.745255	4.277692	H	-1.731897	-2.374975	3.776109
C	0.430573	3.526824	3.549415	C	1.002437	3.612614	3.185927	C	-2.327608	-3.849609	2.299032
H	0.728070	4.033218	4.470192	H	1.254191	4.193248	4.076891	H	-2.881567	-4.461274	3.015595
H	-0.631485	3.286377	3.611603	H	-0.070376	3.707147	3.008032	H	-1.323597	-4.265882	2.198380

H	0.581709	4.203399	2.703477	H	1.536800	4.021419	2.323565	H	-2.823032	-3.895250	1.324944
O	-3.980627	1.102553	-1.013554	O	-4.020241	1.003130	-0.865383	O	4.238336	-0.946072	0.082828
H	-4.088079	0.923813	-1.958091	H	-3.936546	1.036875	-1.829032	H	4.497145	-0.786937	-0.836528
H	-4.228783	2.027177	-0.862387	H	-4.339447	1.874617	-0.584653	H	4.553792	-1.834449	0.309478
N	1.169959	-3.850266	2.978652	N	1.398777	-4.181830	2.424897	N	-1.340053	3.967220	2.783059
H	1.136041	-4.765790	3.435634	H	1.352686	-5.149159	2.756954	H	-1.184194	4.616838	3.559427
H	1.627254	-3.196454	3.621700	H	1.981270	-3.654188	3.081596	H	-2.256697	3.527421	2.910779
H	1.770297	-3.914593	2.149785	H	1.884534	-4.163658	1.521916	H	-1.398794	4.513620	1.918636
O	-3.069018	0.477456	2.672719	O	-2.746933	-0.095620	2.957924	O	2.006764	-0.740890	3.293812
H	-3.760947	-0.112326	3.003653	H	-3.637879	-0.060800	3.333113	H	2.790367	-0.777430	3.860244
C	-1.083871	-3.161987	-4.082828	C	-1.194112	-3.036685	-4.106361	C	2.215404	3.569158	-3.110178
H	-1.585283	-4.049015	-4.477388	H	-1.694490	-3.929569	-4.488797	H	2.731409	4.529746	-3.179736
H	-0.008740	-3.347730	-4.074826	H	-0.116061	-3.195834	-4.155852	H	1.196335	3.697494	-3.478950
H	-1.284901	-2.313549	-4.743315	H	-1.449351	-2.183903	-4.741911	H	2.727547	2.841406	-3.746210
O	1.110026	-1.929346	1.210331	O	1.249490	-2.046553	0.907128	O	-1.613633	2.184431	0.885324
O	-0.804226	2.057016	-0.938242	O	-1.170972	2.205604	-0.733461	O	1.788789	-2.272968	-1.212168
N	-1.025871	4.215373	-2.398469	N	-1.379325	4.345051	-2.245837	N	1.399593	-3.178119	-3.653326
H	-1.860446	3.647693	-2.217657	H	-2.173283	3.724659	-2.051809	H	1.961121	-2.455637	-4.113201
H	-1.230720	4.869520	-3.158918	H	-1.591913	4.911583	-3.071604	H	1.139473	-3.866836	-4.364926
H	-0.852951	4.765371	-1.551395	H	-1.299051	4.981185	-1.447044	H	2.005558	-3.625974	-2.954579
C	1.447108	4.132465	-2.749767	C	1.122852	4.399087	-2.284747	C	-0.872369	-3.674915	-2.733955
H	1.653047	4.579204	-1.772882	H	1.181539	4.835859	-1.283182	H	-0.505434	-4.457104	-2.064010
H	1.403334	4.923436	-3.502017	H	1.130640	5.200936	-3.026317	H	-1.171175	-4.125412	-3.683808
H	2.267654	3.459892	-3.001429	H	2.003015	3.773946	-2.439768	H	-1.752932	-3.209237	-2.289087
C	-1.033189	-3.024897	3.828222	C	-0.546959	-3.223429	3.667755	C	-0.132173	2.175021	4.035126
H	-1.159112	-3.900486	4.469059	H	-0.663357	-4.120392	4.280067	H	0.117338	2.862673	4.846685
H	-2.018985	-2.687147	3.506331	H	-1.525716	-2.761230	3.532736	H	0.666711	1.436137	3.952909
H	-0.567030	-2.222452	4.407272	H	0.098586	-2.512789	4.193306	H	-1.062816	1.655561	4.281024
C	-0.056079	-2.207724	1.600591	C	0.113031	-2.354022	1.361061	C	-0.539109	2.013364	1.518761
H	-2.373688	0.498143	3.347421	H	-2.142680	0.255775	3.627788	H	1.306942	-1.246202	3.733519
$\{[Nd(H_2O)_4(L\text{-}Ala)_2]_2\}^{6+}$ septet 6-31G**(C,N,O,H)/PCM(H_2O)											
Nd	-1.820376	0.580929	-0.973167								
Nd	1.784941	-0.710888	0.779164								
N	-3.591417	-1.753506	2.954854								
H	-3.586571	-1.012042	3.660701								
H	-4.310521	-2.430659	3.223340								
H	-3.849815	-1.317821	2.058912								
N	3.552376	2.884125	-1.092247								
H	4.061134	3.770299	-1.037552								
H	4.096553	2.243374	-1.677038								
H	3.514733	2.458045	-0.145999								
O	2.046516	0.765051	-1.104606								
O	-2.078089	-0.816740	1.011747								
O	-3.398788	-1.265528	-1.630346								
H	-3.733693	-1.885187	-0.966372								
H	-4.022479	-1.290896	-2.370027								
O	-0.120785	-1.165403	-1.046213								
O	3.415154	1.278095	1.400005								
H	4.299146	0.873508	1.415277								
H	3.319280	1.768172	2.230860								
O	0.331220	1.145122	1.214823								
O	2.739060	-2.995371	1.333467								

H 2.498136 -3.686319 0.698599<br>H 2.700278 -3.398439 2.212176<br>O -0.168938 -1.670313 1.874093<br>O -2.402317 2.805179 -1.990011<br>H -1.628143 3.382514 -2.067581<br>H -3.105002 3.322820 -1.572214<br>O 0.155878 1.820525 -1.733237<br>O -4.058361 1.330415 -0.129068<br>H -4.863121 0.796404 -0.184468<br>H -4.071783 1.773655 0.731561<br>C -1.416800 -1.538458 1.812790<br>C 1.391957 1.772169 -1.490136<br>C 0.642297 -1.980646 -1.657673<br>C 2.168402 3.084432 -1.659395<br>H 1.687493 3.835821 -1.028325<br>C -0.217084 2.936784 2.700540<br>H 0.710479 3.474804 2.490472<br>C 0.153768 -2.610201 -2.960488<br>H -0.243535 -1.826253 -3.607074<br>O -1.480579 0.221680 -3.467696<br>H -0.829364 0.848505 -3.818139<br>H -2.194712 0.185333 -4.120487<br>C -2.227720 -2.389458 2.804393<br>H -1.747655 -2.358797 3.782571<br>C -2.355510 -3.831436 2.308938<br>H -2.914413 -4.437316 3.026551<br>H -1.354644 -4.255503 2.209699<br>H -2.850767 -3.875367 1.334710<br>O 4.228508 -0.968428 0.073099<br>H 4.498716 -0.786201 -0.838511<br>H 4.531600 -1.866011 0.279445<br>N -1.302597 3.984925 2.777122<br>H -1.144798 4.626093 3.560072<br>H -2.224166 3.552619 2.895521<br>H -1.351156 4.540208 1.917720<br>O 2.002891 -0.751197 3.285533<br>H 2.785680 -0.773075 3.853817<br>C 2.233700 3.566192 -3.107323<br>H 2.753206 4.525078 -3.173687<br>H 1.215477 3.699115 -3.476785<br>H 2.743968 2.838465 -3.744910<br>O -1.602058 2.202511 0.883947<br>O 1.769076 -2.303876 -1.207718<br>N 1.361277 -3.201309 -3.650510<br>H 1.921904 -2.482036 -4.116548<br>H 1.092845 -3.890587 -4.358497<br>H 1.970875 -3.650186 -2.955503<br>C -0.905175 -3.687098 -2.712012<br>H -0.536309 -4.469258 -2.043144<br>H -1.212908 -4.138383 -3.658615<br>H -1.780400 -3.216848 -2.261521<br>C -0.108006 2.180398 4.024302<br>H 0.152546 2.864405 4.835453		

H 0.681896 1.432329 3.938541<br>H -1.043681 1.671582 4.273119<br>C -0.523635 2.025504 1.509243<br>H 1.308045 -1.260938 3.727933		
